# Cryptochrome 1 mediates light-dependent inclination magnetosensing in monarch butterflies

**DOI:** 10.1038/s41467-021-21002-z

**Published:** 2021-02-03

**Authors:** Guijun Wan, Ashley N. Hayden, Samantha E. Iiams, Christine Merlin

**Affiliations:** 1 Department of Biology and Center for Biological Clocks Research, Texas A&M University, College Station, TX USA; 2 Department of Entomology, College of Plant Protection, Nanjing Agricultural University, Nanjing, China; 3 Genetics Interdisciplinary Program, Texas A&M University, College Station, TX USA

**Keywords:** Behavioural ecology, Behavioural genetics

## Abstract

Many animals use the Earth’s geomagnetic field for orientation and navigation. Yet, the molecular and cellular underpinnings of the magnetic sense remain largely unknown. A biophysical model proposed that magnetoreception can be achieved through quantum effects of magnetically-sensitive radical pairs formed by the photoexcitation of cryptochrome (CRY) proteins. Studies in *Drosophila* are the only ones to date to have provided compelling evidence for the ultraviolet (UV)-A/blue light-sensitive type 1 CRY (CRY1) involvement in animal magnetoreception, and surprisingly extended this discovery to the light-insensitive mammalian-like type 2 CRYs (CRY2s) of both monarchs and humans. Here, we show that monarchs respond to a reversal of the inclination of the Earth’s magnetic field in an UV-A/blue light and CRY1, but not CRY2, dependent manner. We further demonstrate that both antennae and eyes, which express CRY1, are magnetosensory organs. Our work argues that only light-sensitive CRYs function in animal light-dependent inclination-based magnetic sensing.

## Abstract (web summary)

Exactly how some animals use magnetic fields to navigate is a longstanding puzzle. A study using a new behavioural assay and transgenic butterflies finds the cryptochrome gene necessary for inclination-based magnetic sensing, and shows that both antennae and eyes, which express this gene, are magnetosensory organs.

## Introduction

The ability of many animals to sense and exploit the Earth’s magnetic field for directional information during long-distance migration^1–3^ underscores the biological importance of this enigmatic sense. Two main models have been proposed to explain its biological basis: a magnetic particle-based process mediated by magnetite crystals functioning as compass needles^4^, and a radical-pair-based process relying on the spin chemistry of radical-pair reactions initiated by light in specialized photoreceptors^5–7^. In the radical-pair hypothesis, a light-induced electron transfer reaction in the photoreceptor generates an unstable radical pair in a singlet (antiparallel) spin state which can evolve to a triplet (parallel) state. The Earth’s magnetic field would affect the singlet-triplet interconversion in an orientation-dependent manner relative to the sensor molecule, leading to a change in the singlet-triplet yield that would in turn trigger a physiological and behavioral response^5,8^. Although in vitro experiments with a synthetic carotenoid–porphyrin–fullerene model compound show that a radical-pair-based chemical compass can operate at the Earth’s strength magnetic field^9^, a radical-pair mechanism for animal magnetoreception has yet to be demonstrated in the relevant photoreceptor.

Due to their photoreceptive function, cryptochrome (CRY) flavoproteins have been proposed as the candidate light-dependent magnetic detectors^6^. Consistent with the theoretical framework of the radical-pair mechanism, CRY1 of the plant *Arabidopsis thaliana* has been shown to form magnetically sensitive radical pairs after photoexcitation of a flavin adenine dinucleotide (FAD) cofactor^10^. In animals, CRYs, which are best known for their role in circadian function, can be classified into three categories: *Drosophila*-like type 1 CRYs, mammalian-like type 2 CRYs, and bird-like type 4 CRYs. Type 1 CRYs (CRY1s) are UV-A/blue-light photoreceptors responsible for the synchronization of the circadian clock to the daily light:dark cycle^11,12^, and are present in most insects but absent in vertebrates. Despite having no known roles in clock function, type 4 CRYs are also light-sensitive^13^. In contrast, type 2 CRYs (CRY2s) are light-insensitive, function as circadian transcriptional repressors^12,14,15^ and are found not only in mammals but also in all insects studied so far with the exception of flies in the brachyceran lineage^12,16,17^. Importantly, genetic and behavioral studies in the fruit fly *Drosophila* provided the first and only in vivo demonstration to date that light-sensitive type 1 CRYs mediates light-dependent magnetoreception in a wavelength-dependent manner^18–21^. Surprisingly, this finding was later extended to type 2 CRYs by showing that monarch butterfly and human CRY2s overexpressed in CRY-deficient flies could restore magnetosensitivity and its light-dependency, albeit with modest responses^21,22^. The discovery that type 2 CRYs mediated light-dependent magnetosensing suggested that they may undergo the necessary photochemical reactions for magnetosensitivity in the fly’s cellular environment^18,21^. How this could be achieved is unclear because unlike CRY1s, CRY2s lack the structural features to bind FAD^23^. Alternatively, these unexpected results could be due to the use of CRYs overexpression and nonphysiological magnetic field intensities (up to ten times stronger than the natural geomagnetic field) in *Drosophila* studies^20,21^. The use of another model that is amenable to genetic manipulations and responds to directional magnetic fields of intensities found on Earth could help genetically re-evaluate the contribution of both types of CRYs in animal magnetoreception.

Here, we show that the migratory monarch butterfly, which possesses not only both types of CRYs^12^ but also a light-dependent inclination compass that may help guide its long-distance migration^2^, can be used as a model system to dissect the molecular mechanisms underlying magnetoreception. Using an integrated approach combining behavior and CRISPR/Cas9-mediated targeted mutagenesis, we demonstrate that monarchs respond to a reversal of the inclination of the Earth’s magnetic field in a UV-A/blue light- and CRY1-dependent manner, and that both antennae and eyes, which express CRY1, are magnetosensory organs. Importantly, we also show that CRY2 is dispensable to the light-dependent inclination-based magnetic sensing abilities of monarchs, challenging the idea that mammalian-like CRY2s contribute to light-dependent animal magnetoreception. By providing evidence that the CRY1 protein is involved in the detection of vector direction (i.e., magnetic inclination), which supports its role in a geomagnetic compass, this work has important implications to understand how CRY1-based magnetoreception is achieved at a mechanistic level.

## Results

### Individual-level behavioral assay for light-dependent magnetoreception in monarchs

**Fig. 1:**
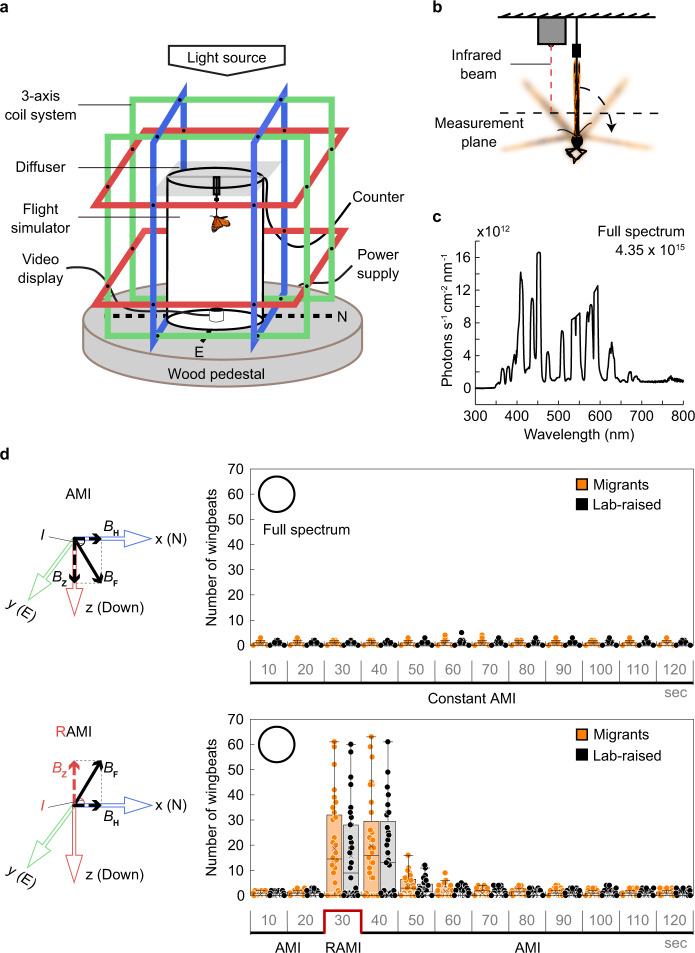
A robust individual-level behavioral assay for light-dependent magnetoreception in the monarch butterfly. **a** Monarchs are tethered and fixed in the center of a flight simulator surrounded by a 3D Helmholtz coil system, facing the geomagnetic South. Of the three pairs of coils (red, blue, and green), the plane of the green pair is parallel to geomagnetic North (N) and perpendicular to geomagnetic east (E). **b** Frontal view of the tethered monarch and the infrared beam system counting the number of wingbeats. **c** Irradiance curve of full-spectrum light conditions (~350–800 nm) during trials. **d** Left panels, North-East-down coordinate system of the ambient magnetic inclination (AMI) and the reversal of AMI (RAMI). *B*_F_, *B*_H_, *B*_Z_, and *I* represent the geomagnetic vector, horizontal component, vertical component, and inclination of the magnetic field, respectively. Right panels, magnetic responses of wild-caught migratory (orange) and laboratory-raised (black) wild-type monarchs to a reversal of magnetic inclination (red line) under full spectrum light (white circle) (*n* = 32 for each group). Each box plot shows the number of wingbeats for every 10 s time bin (median, centerline; interquartile range (IQR), box; 1.5× IQR, whiskers). Each dot represents the response of an individual. Statistical significance between wild-caught migrants and laboratory-raised monarchs was tested for each time bin using a two-tailed Mann–Whitney *U* test at *p* < 0.05. No significance was found.

Fall migratory monarch butterflies have been shown to orient to the magnetic inclination when tested in a flight simulator indoors^2^. However, the overall low success rate (of ~23%^2^), associated with the fact that monarchs have to fly continuously for at least 5 min for proper quantification of oriented behavior^24^, did not favor the use of an orientation-based assay for genetic analyses. We thus began our study by developing a behavioral assay that would rapidly assess responses of a laboratory-raised individual monarch to a change in Earth’s strength magnetic field under different lighting conditions. Given that fall migratory monarchs switch their flight orientation by ~180° in response to a reversal of magnetic inclination^2^, we explored the possibility that monarchs suspended in a flight simulator but unable to move in the horizontal plane may become hyperactive and show an increase in wingbeat when subjected to a reversal of the ambient magnetic inclination (RAMI). Both fall migrants and wild-type laboratory-raised monarchs were placed individually in a flight simulator surrounded by a custom-built three-axis Helmholtz coil system used to manipulate the three different magnetic field parameters (declination, inclination, and intensity) (Fig. 1a). An infrared beam was mounted next to the butterfly to record and quantify the number of wingbeats (Fig. 1b), and a diffuse full-spectrum white light source of wavelengths and intensity similar to those previously reported for monarch magnetoreception^2^ (~350–800 nm; light intensity: 4.35 × 10^15^ photons s^−1^ cm^−2^; Fig. 1c) was used to illuminate the butterfly from above (Fig. 1a). Each individual was acclimated in darkness for at least 30 min prior to the test before being subjected to 2 min of constant local AMI (control) under white light. After 5 min of break under the same lighting and magnetic conditions, the same individual was subjected again to 2 min of the ambient magnetic field but during which the inclination was reversed for 10 s starting at 20 s after AMI was initiated (RAMI; Fig. 1d). We found that while wild-caught fall migrants and laboratory-raised monarchs exposed to constant AMI of natural geomagnetic field intensity did not show any significant hyperactivity, the same individuals displayed a significant increase in wingbeat upon reversal of the magnetic inclination (RAMI) (Fig. 1d; *p* < 0.001; two-tailed Mann–Whitney *U* test). We termed this behavior magnetic hyperactivity (MH). Consistent with the notion that monarchs sense and orient to the magnetic inclination^2^, we observed that both the AMI to RAMI and RAMI to AMI transitions elicited an increase in wingbeat for the following 10 s, with the response extinguishing rapidly upon return to constant AMI (Fig. 1d). Importantly, the behavioral response observed in laboratory-raised monarchs was indistinguishable from that of wild-caught fall migrants (*p* ≥ 0.5, two-tailed Mann–Whitney *U* test; Fig. 1d), thus opening avenues to genetically dissect the molecular bases of light-dependent magnetoreception at Earth’s strength geomagnetic intensity, as previously suggested^2^.

### *DpCry1*, but not *dpCry2*, is necessary for monarch light-dependent magnetoreception

**Fig. 2:**
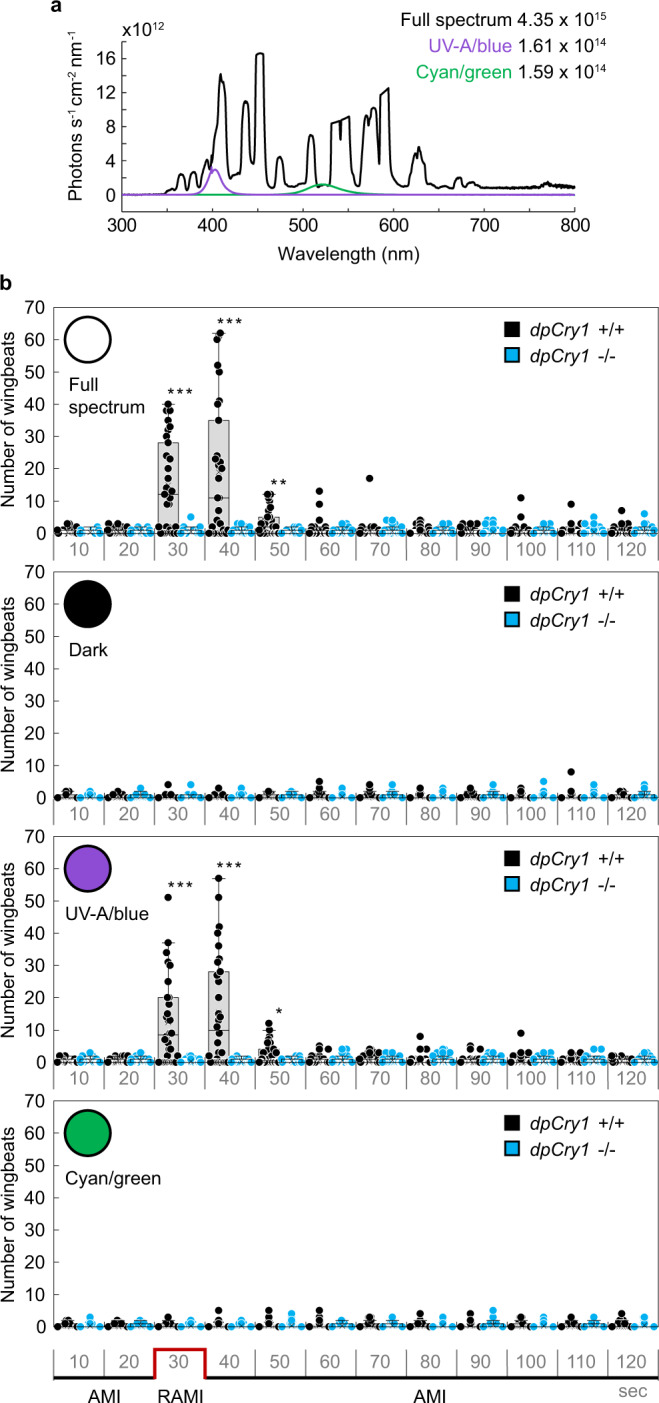
*DpCry1* is necessary for light-dependent magnetoreception in monarchs. **a** Irradiance curves of full-spectrum (~350–800 nm; black line), UV-A/blue (~380–430 nm; violet line) and cyan/green (~480–580 nm; green line) light. **b** Magnetic responses of *dpCry1*^*+/+*^ (black) and *dpCry1*^−*/−*^ (blue) monarchs to a reversal of magnetic inclination (RAMI; red line) under different lighting conditions (full spectrum, white circle; darkness, black circle; UV-A/blue, violet circle; cyan/green, green circle). Each box plot shows the number of wingbeats for every 10 s time bin (median, centerline; interquartile range (IQR), box; 1.5× IQR, whiskers). Each dot represents the response of an individual (*n* = 30 for each genotype). In each lighting condition, statistical significance between genotypes was tested for each time bin using a two-tailed Mann–Whitney *U* test at *p* < 0.05 (Full spectrum: ^**^p = 0.003 for fifth 10 s time bin, ^***^p = 4.757E−7 and *p* = 7.000E−6 for third and fourth 10 s time bins respectively; UV-A/blue: ^*^p = 0.038 for fifth 10 s time bin, ^***^p = 2.000E−6 and *p* = 2.000E−5 for third and fourth 10 s time bins, respectively).

**Fig. 3:**
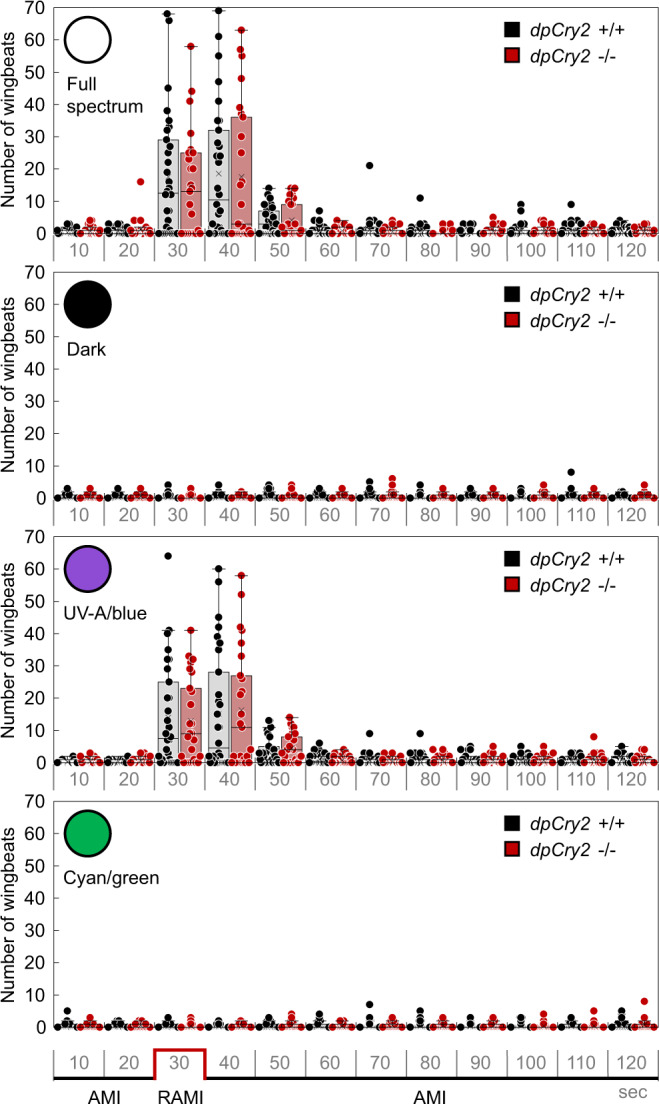
*DpCry2* is dispensable for light-dependent magnetoreception in monarchs. Magnetic responses of *dpCry2*^*+/+*^ (black) and *dpCry2*^*−/−*^ (red) monarchs to a reversal of magnetic inclination (RAMI; red line) under different lighting conditions (full spectrum, white circle; darkness, black circle; UV-A/blue, violet circle; cyan/green, green circle). Each box plot shows the number of wingbeats for every 10 s time bin (median, centerline; interquartile range (IQR), box; 1.5× IQR, whiskers). Each dot represents the response of an individual (*n* = 30 and 25 for *dpCry2*^*+/+*^ and *dpCry2*^*−/−*^, respectively). In each lighting condition, statistical significance between genotypes was tested for each time bin using a two-tailed Mann–Whitney *U* test at *p* < 0.05. No significance was found.

We next used this assay in combination with reverse genetics to genetically re-evaluate the reported function of both light-sensitive *Drosophila*-like CRY1 and light-insensitive mammalian-like CRY2 in mediating light-dependent magnetoreception^20–22^. Using CRISPR/Cas9, we generated a monarch dpCRY1 mutant bearing a 2-bp deletion in the fourth exon of *dpCry1* (Supplementary Fig. 1a). This mutation leads to the introduction of a premature stop codon that generates a truncated protein lacking the functional C-terminal domain that contains the four Trp residues forming a Trp tetrad thought to be necessary for electron transfer and the formation of radical pairs^25–27^ (Supplementary Fig. 1b). Molecular characterization of the truncated mutant revealed a 90% reduction in mRNA expression compared to the wild-type *dpCry1* transcript, suggestive of nonsense-mediated mRNA decay (Supplementary Fig. 1c). In addition, a FLAG-tagged truncated dpCRY1 protein expressed in the DpN1 monarch specific cell line^28^ was undetectable, in contrast to the full-length FLAG-tagged dpCRY1 protein (Supplementary Fig. 1d). Together, these results suggest that the homozygous *dpCry1* mutant monarchs do not express a functional dpCRY1. Homozygous *dpCry1* mutants and wild-type siblings were subjected to our behavioral paradigm of 2 min of control AMI followed by the RAMI treatment under four consecutive lighting conditions (white light, darkness, UV-A/blue, cyan/green; Fig. 2) to test for both light dependence and wavelength dependence of the monarch magnetic response. UV-A/blue light (~380–430 nm; light intensity: 1.61 × 10^14^ photons s^−1^ cm^−2^) and cyan/green light (~480–580 nm: light intensity: 1.59 × 10^14^ photons s^−1^ cm^−2^) were tested using high-power light-emitting diodes (LED) at light intensities close to the ones emitted by the full spectrum light at these spectral ranges (Fig. 2a). The UV-A/blue LED was chosen because the previously reported light dependence of both the monarch inclination compass^2^ and monarch dpCRY1 protein for rescuing magnetosensitivity in CRY-deficient *Drosophila*^20^ was between 380 and 420 nm, while the cyan/green LED was used as a control. As expected, neither *dpCry1*^*+/+*^ nor *dpCry1*^*−/−*^ monarchs showed MH in control AMI conditions regardless of the lighting condition used (Supplementary Fig. 2; Supplementary Videos 1 and 2). Consistent with the fact that the inclination compass of monarchs operates within the UV-A/blue light spectral range^2^, we found that *dpCry1*^*+/+*^ monarchs displayed MH upon AMI to RAMI and RAMI to AMI reversals under both full-spectrum and UV-A/blue light (3^rd^ and 4^th^ 10-s time bin: *p* < 0.001, two-tailed Mann–Whitney *U* test; Fig. 2b), but not in complete darkness or under cyan/green light (*p* ≥ 0.407, two-tailed Mann–Whitney *U* test; Fig. 2b; Supplementary Video 1). In contrast, the MH response to AMI to RAMI and RAMI to AMI transitions was abolished in *dpCry1*^*−/*^^−^ siblings under both full-spectrum and UV-A/blue light (3rd–5th 10-s time bin: *p* ≤ 0.038 between *dpCry1*^*+/+*^ and *dpCry1*^*−/*^^−^ during the 3rd–5th 10-s time bin of treatment, two-tailed Mann–Whitney *U* test; Fig. 2b; Supplementary Video 2). To unambiguously exclude the possibility that the response to RAMI with our single wrapped coils may have been elicited by heat or vibration while injecting current into the coils, we repeated the experiments with a similar three-axis Helmholtz system harboring double wrapped coils. The coils and behavioral apparatus were placed inside a Faraday cage, and the experimenter was blind to the genotypes to preclude any possible subconscious bias. Similar to that observed using single wrapped coils, neither *dpCry1*^*+/+*^ nor *dpCry1*^*−/−*^ monarchs showed MH in control AMI conditions, and the MH observed in *dpCry1*^*+/+*^ monarchs upon AMI to RAMI and RAMI to AMI reversals under both full-spectrum and UV-A/blue light was abolished in *dpCry1*^*−/*^^−^ monarchs (3rd–5th 10-s time bin: *p* ≤ 0.001, 6th 10-s time bin: *p* ≤ 0.05 under full-spectrum and *p* ≤ 0.01 under UV-A/blue light, two-tailed Mann–Whitney *U* test; Supplementary Fig. 3). Importantly, the lack of MH observed in *dpCry1*^−^^*/−*^ monarchs was not due to impaired general activity levels, as these mutants flew as actively in a flight mill as their wild-type siblings over three days (Supplementary Fig. 4; *p* ≥ 0.06, two-tailed Mann–Whitney *U* test). Together, these results demonstrated that the UV-A/blue light-activatable dpCRY1 is necessary for the inclination-based magnetic sense of monarchs exposed to Earth’s strength magnetic fields. The complete lack of MH response to AMI to RAMI and RAMI to AMI reversals observed in *dpCry1*^*−/*−^ monarchs, in which dpCRY2 is intact, also suggested that dpCRY2 was not contributing to the inclination-based magnetic sense of monarchs when exposed to a physiological magnetic field intensity. To genetically confirm this surprising result, we subjected a previously generated *dpCry2* mutant to the same behavioral paradigm as the *dpCry1* mutants. The *dpCry2* mutant harbors a 4-bp deletion in exon 2 that does not significantly impair the expression level of the mRNA compared to wild-type but introduces a premature stop codon (Supplementary Fig. 5a–c) that leads to a truncated protein lacking the C-terminal domain containing the presumptive Trp tetrad^14^. In line with our prediction, we found that *dpCry2*^*−/*^^−^ mutants responded to a reversal of the magnetic inclination under full-spectrum and UV-A/blue light as robustly as *dpCry2*^*+/+*^ monarchs when tested non blindly with the single wrapped coil system (3rd–5th 10-s time bin under both full-spectrum and UV-A/blue light: *p* ≥ 0.5, two-tailed Mann–Whitney *U* test; Fig. 3; Supplementary Fig. 6), as well as when tested blindly with respect to genotypes with the double-wrapped coil system (3rd–6th 10-s time bin under both full-spectrum and UV-A/blue light: *p* ≥ 0.4, two-tailed Mann–Whitney *U* test; Supplementary Fig. 7). In agreement with the function of vertebrate-like CRY2s as vestigial flavoproteins that lack the structural features to bind FAD^23^, our data provide in vivo genetic evidence that, under physiological magnetic field intensity, dpCRY2 does not play a role in the light-dependent inclination-based magnetic sense of monarchs.

### The antennae and the compound eyes are necessary for monarch light-dependent magnetoreception

**Fig. 4:**
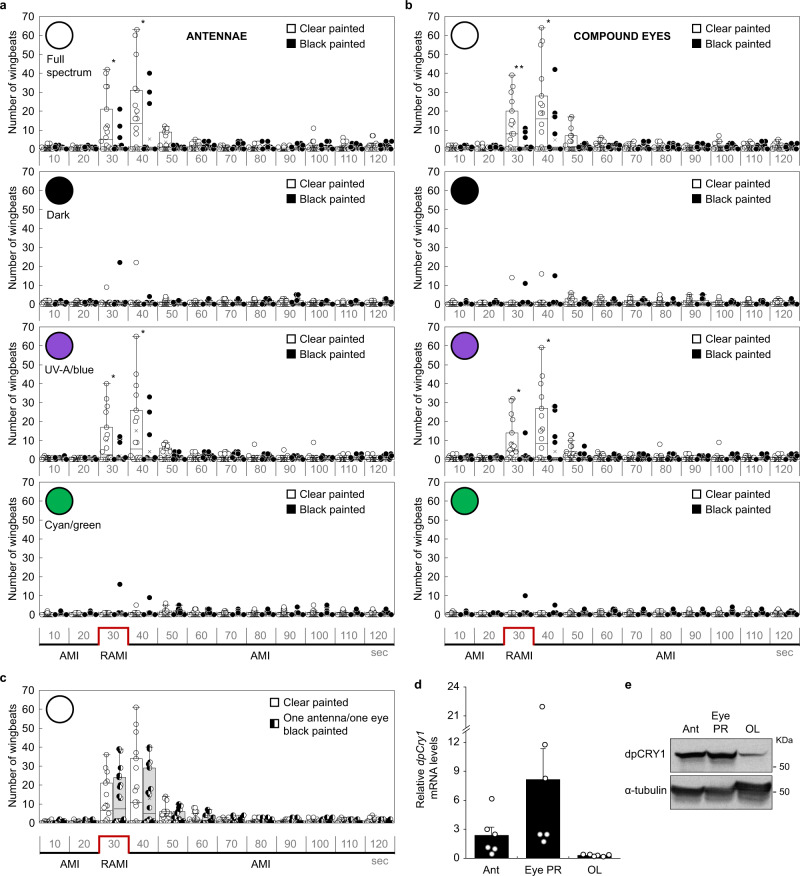
Monarch light-dependent magnetoreception involves both the antennae and the compound eyes. Magnetic responses of wild-type monarchs with black (black bars) and clear (white bars) painted antennae **a** or eyes **b** to a reversal of magnetic inclination (RAMI; red line) under different lighting conditions (full spectrum, white circle; darkness, black circle; UV-A/blue, violet circle; cyan/green, green circle). **c** Magnetic responses of wild-type monarchs with one antenna and one eye painted black (black bars) to a RAMI (red line) under full-spectrum light. Monarchs with clear painted antennae and eyes (white bars) were used as controls. Each box plot shows the number of wingbeats for every 10 s time bin (median, centerline; interquartile range (IQR), box; 1.5× IQR, whiskers). Each dot represents the response of an individual (*n* = 18 for each painting group). Statistical significance between painting groups was tested for each time bin using a two-tailed Mann–Whitney *U* test at *p* < 0.05 (Full spectrum in (**a**): ^*^ *p* = 0.019 and *p* = 0.029 for third and fourth 10 s time bins respectively; UV-A/blue in (**a**): ^*^ *p* = 0.029 and *p* = 0.017 for third and fourth 10 s time bins, respectively; Full spectrum in (**b**): ^*^ *p* = 0.016 for fourth 10 s time bin and ^**^p = 0.009 for third 10 s time bin; UV-A/blue in (**b**): ^*^ *p* = 0.011 and *p* = 0.027 for third and fourth 10 s time bins, respectively). Transcript (**d**) and protein (**e**) expression levels of dpCRY1 in antennae (Ant), compound eye photoreceptors (Eye PR), and optic lobe (OL) of adult monarchs (*n* = 6 per tissue in (**d**)). **d** Data are presented as mean values ± SEM.

Locating the organ(s) in which the magnetic sense operates is critical for a continued dissection of the molecular and neural bases of this enigmatic sense. Previous work has shown that the magnetosensor likely resides in monarch antennae^2^. The contribution of the compound eyes in magnetosensing, which has been shown in the cockroach and *Drosophila*^19,29^, had never been tested in the monarch. We tested the role of both organs in the monarch light-dependent inclination-based magnetic responses by blocking the light input to each organ, individually or in combination, with a nontoxic black paint^2,30^. Monarchs with clear painted organs were used as controls. Consistent with the previously reported role of monarch antennae in the inclination-based magnetic compass^2^, wild-type monarchs with black painted antennae showed a significantly reduced MH response compared to monarchs with clear painted antennae upon AMI to RAMI and RAMI to AMI reversals under both full-spectrum and UV-A/blue light (3rd and 4th 10-s time bins: *p* ≤ 0.03, two-tailed Mann–Whitney *U* test; Fig. 4a; Supplementary Fig. 8a). Interestingly, blocking the light input to the eyes had a similar effect, as monarchs with black painted eyes also exhibited an impaired response to AMI to RAMI and RAMI to AMI reversals compared to monarchs with clear painted eyes under full-spectrum (3rd and 4th 10-s time bin: *p* ≤ 0.02, two-tailed Mann–Whitney *U* test) and UV-A/blue light (3rd and 4th 10-s time bin: *p* ≤ 0.03, two-tailed Mann–Whitney *U* test; Fig. 4b; Supplementary Fig. 8b). While a few outliers still presented an MH response in each of these treatments, their number was reduced with both organs painted black (Supplementary Fig. 9). As previously proposed in *Drosophila*^19^, these results indicated that both antennae and eyes are necessary for monarch magnetosensing and that impairing magnetosensitivity in one organ cannot be compensated by the other. We also wondered whether blocking the light input to a single antenna and a single eye would affect the ability of monarchs to sense and respond to the reversal of the magnetic inclination. We found that monarchs with a single antenna and a single eye painted black showed an MH response that was not significantly different from that of monarchs with both organs painted clear under full-spectrum light (3rd and 4th 10-s time bins: *p* ≥ 0.67, two-tailed Mann–Whitney *U* test; Fig. 4c), irrespective of whether the black painted organs were ipsilateral or contralateral (Supplementary Fig. 10). Not surprisingly, we found that the *dpCry1* transcript and corresponding protein are expressed at relatively high levels in both antennae and the photoreceptor layer of the compound eyes, as compared to the optic lobe (Fig. 4d, e). Taken together, our data provide a genetic demonstration that, under physiological magnetic field intensity, the light-sensitive *Drosophila*-like dpCRY1, but not the mammalian-like dpCRY2, mediates the UV-A/blue light-dependent inclination-based magnetic sense of monarchs through both antennae and eyes.

## Discussion

For more than a decade, *Drosophila* has remained the only organism in which compelling evidence for the involvement of the UV-A/blue light-sensitive type 1 CRYs in animal magnetoreception has been provided^18–21,31^. However, because these studies relied on behavioral assays that used non-directional and nonphysiological magnetic field conditions, a clear demonstration that type 1 CRYs could function in a geomagnetic compass was still missing. By establishing a behavioral paradigm in the monarch, a migratory species that shows robust responses to a change in the inclination of Earth’s strength magnetic field, our work extends the role of type 1 CRYs in UV-A/blue light-dependent magnetoreception to another insect species. It also provides evidence that the CRY1 protein is involved in the detection of vector direction (i.e., magnetic inclination), supporting its role in a geomagnetic compass. Whether the CRY1-mediated inclination magnetic compass of monarchs acts in concert with or as a backup mechanism of the time-compensated sun compass to provide migratory directional information, or whether it is used for positional information (i.e., provides a “map” sense) during their migration remains to be determined^2^.

Importantly, our results also challenge the idea that mammalian-like type 2 CRYs contribute to light-dependent animal magnetoreception. Although monarch and human CRY2s have been shown to mediate light-dependent magnetosensitivity in the *Drosophila* cellular environment^18,21,22^, whether this would be the case in the relevant cellular environment has remained an open question. Studies in the mammalian suprachiasmatic nucleus (SCN), the brain structure harboring the master circadian clock, found no evidence of mammalian CRYs’ involvement in magnetosensing. However, it is unclear whether the SCN is the most appropriate cellular location for such studies^18^. The use of full-body loss-of-function mutants in our in vivo study unambiguously demonstrates that, in the proper cellular environment, monarch CRY2 does not play a role in magnetosensing when exposed to Earth’s strength magnetic field. The discrepancy between our results and a previous study reporting that CRY2 mediates sensitivity to the magnetic declination in cockroaches^29^ could be due to differences in the behavioral assays, as the one used in cockroaches showed only modest effects even in control conditions. Together, our findings support the idea that only light-sensitive CRYs, the sole molecules experimentally proven so far to form radical pairs after photoexcitation^10^, function in animal magnetoreception.

Future work will be necessary to understand how type 1 CRY-based magnetoreception is achieved at a mechanistic level and whether it occurs through a radical-pair mechanism. The formation of radical pairs by CRY has long been thought to occur via a conventional Trp-triad^8^, but genetic studies in *Drosophila* have argued against this possibility^18,19,21^. This is likely due to the fact that the electron transfer instead occurs through a recently discovered Trp tetrad^25,26^. Developing transposon-based transgenic approaches, which should be readily achievable in the monarch, should allow us to test this possibility. Despite the central role that light-sensitive CRYs play in light-dependent magnetoreception, the notion that they function as bona fide photo-magnetoreceptors is still under debate^32^. The absence of MH to a reversal of the ambient magnetic field under green light emitting between 480 and 580 nm in our assay may provide some insights. Indeed, the absorption spectra of type 1 CRYs bound to fully oxidized FAD necessary for radical pair formation extends to ~500 nm^33^. The lack of magnetic response under green light could be due to insufficient photo-activation to give rise to radical pair formation in CRY1 between 480 and 500 nm. However, if there was sufficient photo-activation of CRY1, the lack of magnetic responses in our experiments could be interpreted as evidence for another photoreceptor in light-dependent magnetoreception with an absorption spectrum that extends less far into the green than does CRY1s. A UV opsin would be an obvious candidate. The potential involvement of opsins in magnetoreception is not without precedent, as direct interaction between a member of the opsin family and the avian type 4 CRY has recently been reported^34^, and could be genetically tested in the monarch. Finally, our findings that antennae and eyes are both necessary for monarch magnetoreception also open avenues of investigations into the neural substrates in which CRY operates either as a photomagnetoreceptor or as a signaling molecule acting downstream of the magnetoreceptor. More broadly, this study highlights how the advent of genome editing in unconventional organisms with more relevant biology than conventional model systems can advance mechanistic, genetic investigations of behavior.

## Methods

### Monarch butterfly rearing and housing

Laboratory-raised monarch butterflies were raised from eggs laid on milkweed plants (*Asclepias curassavica*). Upon eclosion, larvae were fed cuttings until the second instar before being transferred onto a semi-artificial diet and reared individually until adult eclosion under a 15-h light: 9-h dark (LD) cycle at 25 °C and 70% humidity^14^. After emergence, adults were kept in glassine envelopes under the same lighting and humidity conditions at 21 °C. Adult fall migrants were captured by Dale Clark on 17 October and 23 October 2018 in Dallas, Texas (latitude 32.77° N, longitude 96.79° W) and by members of the laboratory between 19 October and 27 October in College Station, Texas (latitude 30.62° N, longitude 96.33° W). Wild-caught monarchs were housed indoors in glassine envelopes in an 11-h light: 13-h dark (LD) cycle set to prevailing light conditions. Laboratory-raised and wild-caught adult monarchs were fed a 25% honey solution every other day.

### Behavioral apparatus

Monarchs were tethered via a tungsten rod glued to their thorax and fixed in the center of a flight simulator surrounded by a 106 cm × 106 cm × 106 cm custom-built three-axis Helmholtz coils system and placed in the center of a large room maintained at ~21 °C. Diffuse illumination was provided from above by either a full spectrum white lamp (400 W metal halide lamp; iPower; 50% dimmed; spectrum: peak at 453 nm, range: 350–800 nm, intensity: 4.35 × 10^15^ photons s^−1^ cm^−2^), a UV-A/blue LED (LED Engin; LZ1-10UB00-01U7; spectrum: peak at 399 nm, range: ~380–430 nm, intensity: 1.61 × 10^14^ photons s^−1^ cm^−2^), or a cyan/green LED (LED Engin; LZ1-00G102-0G23; spectrum: peak at 520 nm; range: ~480–580 nm; light intensity: 1.59 × 10^14^ photons s^−1^ cm^−2^) (Fig. 2a), and a light diffuser placed directly on top of the opening of the modified flight simulator^2^. The Helmholtz coils were set up with the plane of the blue pair of coils parallel to the geomagnetic north (N) and perpendicular to the geomagnetic east (E) and the monarch head facing the geomagnetic south (Fig. 1a). An infrared beam was mounted to the transparent acrylic arm next to the aluminum rod on which the monarch was suspended to record the number of wing beats. The infrared ray emitted was parallel to the aluminum rod and perpendicular to the measurement plane and the beam was broken when the wing opening was at minimum ~45° from the closed position, generating two breaks for each wing beat. A camera hooked to a recording system was placed underneath the fixed monarch to discriminate between active flight and possible basking behavior (i.e., occasional outstretching of wings) (Fig. 1a, b). Light parameters were measured with a Jaz-ULM-200 spectrometer (Ocean Optics) inside the flight simulator with the light diffuser in place and at the level of the monarch’s head. The full spectrum and UV-A/blue lights used in this study provided monarchs with light wavelengths overlapping with the ones reported to enable magnetic responses in monarchs^2^ or other insects^19,20,29,35^.

### Assay for magnetic responses under different lighting conditions

Under each lighting condition, individual monarchs were subjected for 2 min to an ambient geomagnetic field naturally occurring in College Station, TX (i.e., the location where the work was performed; latitude 30.62° N, longitude 96.33° W; geomagnetic total vector: *B*_F_ ~44.52 μT, ambient magnetic declination: *D* ~0.1°, and AMI: *I* + 59.1°). The same individual was then subjected to 20 s of constant AMI followed by 10 s of reversal of the magnetic inclination (RAMI; *B*_F_ ~44.52 μT, *D* ~0.1°, and *I* −59.1°) and 90 s of constant AMI. For each monarch tested, this sequence was repeated successively under full-spectrum light, darkness, UV-A/blue light, and cyan/green light. Each monarch tested was acclimated in darkness for at least 30 min prior to the test and between each lighting condition, and for at least 5 min under the tested lighting condition between the AMI control and AMI-RAMI-AMI treatment. To explore the genetic basis of magnetoreception in the monarch butterfly, *dpCry1* and *dpCry2* homozygous mutants and their corresponding wild-type siblings were tested. Both genotypes were tested on the same day, and mutant and wild-type monarchs of a given line were tested in pairs. Monarchs used in the assay were at least 14-days old, were tethered with tungsten rods glued to their thorax at least 3 days before testing their magnetic responses, and were fed a 25% honey solution 1 h before the trials on the day of testing. The magnetic parameters generated by the Helmholtz coils (field intensity, declination, and inclination) were measured throughout the course of the study with a magnetometer (HONOR TOP, Model 191A^35^; Supplementary Fig. 11a–c). Any potential heat effect generated by the coils during the RAMI procedure was excluded by monitoring the temperature at the position of the monarch in the flight simulator with a HOBO Temperature Relative Humidity Data Logger (U10-003; Supplementary Fig. 11d).

### Generation of a monarch *dpCry1* knockout

Monarch *dpCry1* knockouts were generated via CRISPR/Cas9-mediated targeted mutagenesis. The guide RNA (gRNA) was selected to target exon 4 of the 12-exons containing *dpCry1*, and annealed synthetic oligomers (*gRNAOligoCry1_F*, 5′-TAGGGACCTGAACGGCGTCAACTT-3′ and *gRNAOligoCry1_R*, 5′-AAACAAGTTGACGCCGTTCAGGTC-3′; Supplementary Table 1) were subcloned into a DR274 plasmid^36^ at the *BsaI* cleavage site^17,37,38^. In vitro transcription of *Streptococcus pyogenes* Cas9 mRNA was performed from a pCS2-nCas9n plasmid^39^ linearized with *Xba*I and purified with phenol-chloroform using the mMessage mMachine T3 transcription kit (Ambion). The sgRNA was in vitro transcribed using T7 RNA polymerase (Promega) and template polymerase chain reaction (PCR) products amplified from the sgRNA-containing DR274 plasmid with the following primers (*sgRNACry1_F*, 5′-ATTGAGCCTCAGGAAACAGC-3′ and *sgRNACry1_R*, 5′-AAAAGCACCGACTCGGTGCC-3′) and purified with phenol–chloroform. Eggs were collected and microinjected within 20 min of being laid under a dissecting microscope with a mix of Cas9 mRNA and gRNA (at 0.5 µg/µl and 0.25 µg/µl, respectively) loaded along with food coloring into a pulled borosilicate glass needle (World Precision Instruments, Inc.) attached to an IM 300 microinjector (Narishige). Surviving larvae were raised individually as described above. The presence of somatic mutations was assessed by Cas9-based cleavage assays of PCR products flanking the targeted region amplified from genomic DNA of larval sensors using the following primers: *gDNACry1_F*, 5′-CTGGCCTTGATCGCTTACAG-3′ and *gDNACry1_R*, 5′-CGTACTCCACAGCCAATCTC-3′. Purified PCR products (150–200 ng) were incubated for 3 h at 37° with purified a Cas9 protein (100 ng), the sgRNA (100–300 ng) used for targeting, bovine serum albumin (1 μg/μl; New England Biolabs, NEB), and NEB Buffer 3 (1×)^17,37,38^. Reactions were then incubated with 4 μg of RNase A (Amresco) for 2 h at 37° and stopped using a 6× stop solution^40^. Purified products were resolved with agarose gel electrophoresis and EtBr staining. Larvae presenting a high degree of somatic targeting were raised to adulthood, surviving adults of the opposite sex were crossed and their progeny was screened for the presence of mutated alleles as described above. Mutated alleles were sequenced and a two-base pair deletion introducing a premature stop codon was selected to establish a mutant line.

### Genetic crosses

*DpCry1* and *dpCry2* homozygous mutants (^−/−^) and wild-type siblings (^+/+^) were respectively obtained by intercrossing heterozygous males and females of a given line. *DpCry2* mutants were generated previously^14^ and were maintained in the laboratory.

### Painting of compound eyes and antennae

To identify putative magnetosensitive organs, the antennae and/or compound eyes of laboratory-raised wild-type monarchs were covered under a microscope from the tip to the base of the flagellum with either an enamel-based clear paint for control (Model master clear topcoat; Testors no. 2736) or an enamel-based black paint (Glossy black; Testors no. 1147) to prevent light input to the organ^30,37^. The completeness of painting was verified under the microscope both after painting and after the behavioral assay performed 3 days later. The magnetic responses of the three painting groups (i.e., painted antennae, painted eyes, and painted antennae and eyes) were tested on the same day, and for each painted group clear-painted and black painted monarchs were tested in pairs. Any potential toxic effect of the black paint was excluded by performing behavioral assays on wild-type monarchs with one eye and one antenna painted black, either in an ipsilateral or contralateral combination, and monarchs with both antennae and eyes painted clear as controls.

### Real-time qPCR

To test for *dpCry1* expression in the putative magnetosensitive organs tested, antennae, compound eye photoreceptors, and optic lobes were dissected from lab-raised adult wild-type monarchs and stored at −80 °C. Dissections of compound eye photoreceptors and photoreceptor-free optic lobes were performed in 0.5× RNA later (Invitrogen) to avoid RNA degradation. Total RNA from the antennae and eye photoreceptors was extracted using 350 µL of RNA extraction buffer (100 mM Tris pH 7.5, 100 mM LiCl, 20 mM DTT, and 10% sodium dodecyl sulfate (SDS)) followed by purification with acid–phenol–chloroform. Total RNA from the optic lobes was extracted using an RNeasy Mini kit (Qiagen). All RNA samples were treated with RQ1 Dnase (Promega), and reverse transcribed using random hexamers (Promega) and Superscript II Reverse Transcriptase (Thermo Scientific). Quantification of gene expression was performed on a QuantStudio^™^ 6 Flex Real-Time PCR System (Thermo Scientific) using 9 ng of cDNA template, iTaq Universal SYBR Green Supermix (Bio-Rad), and primers. The monarch *dpCry1* and control *rp49* primers were as follows: *cDNACry1*_*F*, 5′-CGAGCACGTCGCACACA-3′; *cDNACry1*_*R*, 5′-TCCTCCATTGGCCTTGATGA-3′; *cDNArp49*_*F*, 5′-TGCGCAGGCGTTTTAAGG-3′; *cDNArp49*_*R*, 5′-TTGTTTGATCCGTAACCAATGC-3′. The near 100% efficiency of *dpCry1* primer set was validated by determining the slope of Ct versus dilution plot on a dilution series. Individual reactions were used to quantify each RNA level in a given cDNA sample, and the average Ct from duplicated reactions within the same run was used for quantification. The data were normalized to *rp49* as an internal control and normalized to the mean of one sample within a set for statistics.

### Western blotting

To test if dpCRY1 was expressed in the putative magnetosensitive organs identified in behavioral experiments, antennae, compound eye photoreceptors, and optic lobes from five adult wild-type monarchs were dissected in Ringer’s solution and flash frozen. Proteins were extracted in 100 μl lysis buffer [150 mM NaCl, 50 mM Tris-Cl, pH 7.4, 0.5% NP40, 1 mM EDTA, 1× Protease Inhibitor Tablet (Pierce)], and concentrations were measured using the Pierce Coomassie Plus Assay kit (Thermo Fisher). For each sample, 3 μg of protein was loaded per lane onto a 7.5% SDS-PAGE. DpCRY1 was detected using a guinea pig anti-dpCRY1 primary antibody (1:500; CRY1-GP37^41^) and a peroxidase affinipure donkey anti-guinea pig IgG secondary antibody (1:1000; Jackson ImmunoResearch #706-035-148). Tubulin was detected using a mouse anti-α tubulin monoclonal antibody (1:10,000; Sigma B-5-1-2) and a goat anti-mouse IgG HRP secondary antibody (1:1000; Invitrogen, 31430).

### Statistical analysis

Data analyses were performed with IBM SPSS Statistics 25. For behavioral data and measurements of magnetic field parameters, normality and equality of variances were performed with the Shapiro–Wilk and Levene’s tests, respectively. Since all behavioral data were either non-normally distributed (*p* < 0.05) and/or nonhomogeneous for variances (*p* < 0.05), a two-tailed nonparametric Mann–Whitney *U* test was used for group comparisons between monarch sources, genotypes, or painting groups for each 10 s time bin under each tested conditions, and between magnetic field parameters. For gene expression data, an unpaired Student’s *t* test was used for group comparisons.

### Reporting summary

Further information on research design is available in the Nature Research Reporting Summary linked to this article.

## Supplementary information

Supplementary Information

Peer Review File

Reporting Summary

Description of Additional Supplementary Files

Supplementary Video 1

Supplementary Video 2

## Data Availability

A reporting summary for this Article is available as a Supplementary Information file. Source data are provided with this paper.

## Source data

Source Data
