# Supplementary material for: Cryptochrome 1 mediates light-dependent inclination magnetosensing in monarch butterflies
Source: Nat Commun. 2021 Feb 3;12:771. doi: 10.1038/s41467-021-21002-z (PMC7859408; doi:10.1038/s41467-021-21002-z)
Supplement: Supplementary file 1 — Supplementary Information [file 41467_2021_21002_MOESM1_ESM.pdf]

Supplementary Information for

Cryptochrome 1 mediates light-dependent inclination magnetosensing in monarch butterflies

Guijun Wan, Ashley N. Hayden, Samantha E. Iiams and Christine Merlin

Supplementary Figures 1-11

Supplementary Figure 12 (Uncropped western blot of Figure 4e)

Supplementary Table 1

Supplementary Methods

Supplementary References

Supplementary Figures

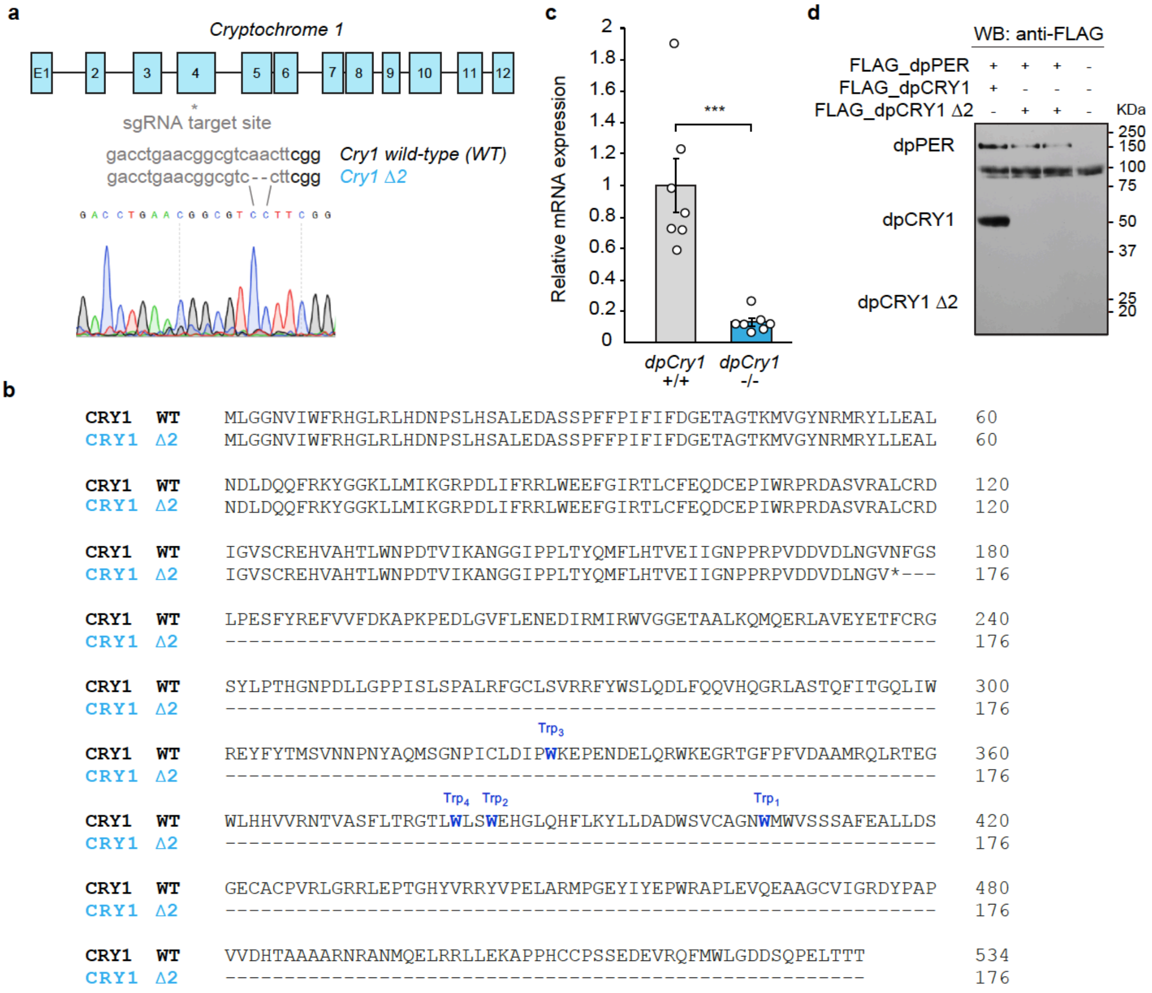

Supplementary Figure 1: *Cryptochrome 1* loss-of-function monarch mutant generated by CRISPR/Cas9-mediated targeted mutagenesis. a, Schematic representation of monarch *dpCry1* genomic locus. The gray star indicates the position of the single guide RNA (sgRNA) used to induce indel mutations. The sgRNA sequence at the target site is shown in gray and is followed by a protospacer adjacent motif (PAM; black). A 2-bp deletion (Δ2; depicted by dashes) in G_1 progeny was used to establish a *dpCry1* loss-of-function mutant line. **b,** Amino acid sequence alignment of wild-type (WT) and truncated loss-of-function (Δ2) dpCRY1 proteins. The four tryptophan (Trp) residues involved in the Trp tetrad are highlighted in dark blue. **c,** Relative *dpCry1* mRNA expression in brains of wild-type (gray) and homozygous *dpCry1Δ2* mutant (blue) monarchs (n = 7 for each genotype). Data are presented as mean values ± SEM. Unpaired Student's t test: ***, $p = 0.0003$. **d,** Western blot (WB) of monarch DpN1 cells transfected with (+) or without (-) FLAG-tagged dpPER and either FLAG-tagged full

length dpCRY1 or FLAG-tagged dpCRY1Δ2 mutant, probed with an anti-FLAG antibody.

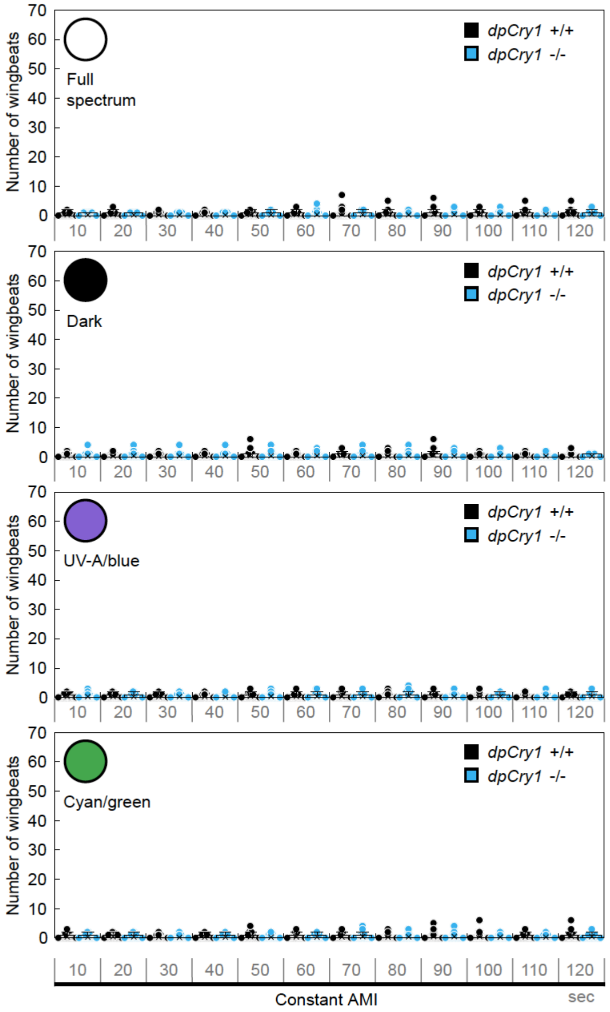

Supplementary Figure 2: Absence of magnetic response in *dpCry1*$^{+/+}$ (black) and *dpCry1*$^{-/-}$ (blue) monarchs subjected to control constant ambient magnetic inclination (AMI) under different lighting conditions. Each box plot shows the number of wingbeats for every 10 seconds (sec) time bin (median, center line; interquartile range (IQR), box; 1.5 × IQR, whiskers). Each dot represents the response of an individual (n = 30 for each genotype). In each lighting condition, statistical significance between genotypes was tested for each time bin using a two-tailed Mann-Whitney U test at $p < 0.05$. Light tested was either full-spectrum (~350 - 800 nm, white circle), UV-A/blue (~380 - 430 nm, violet circle) or cyan/green (~480 - 580 nm, green circle).

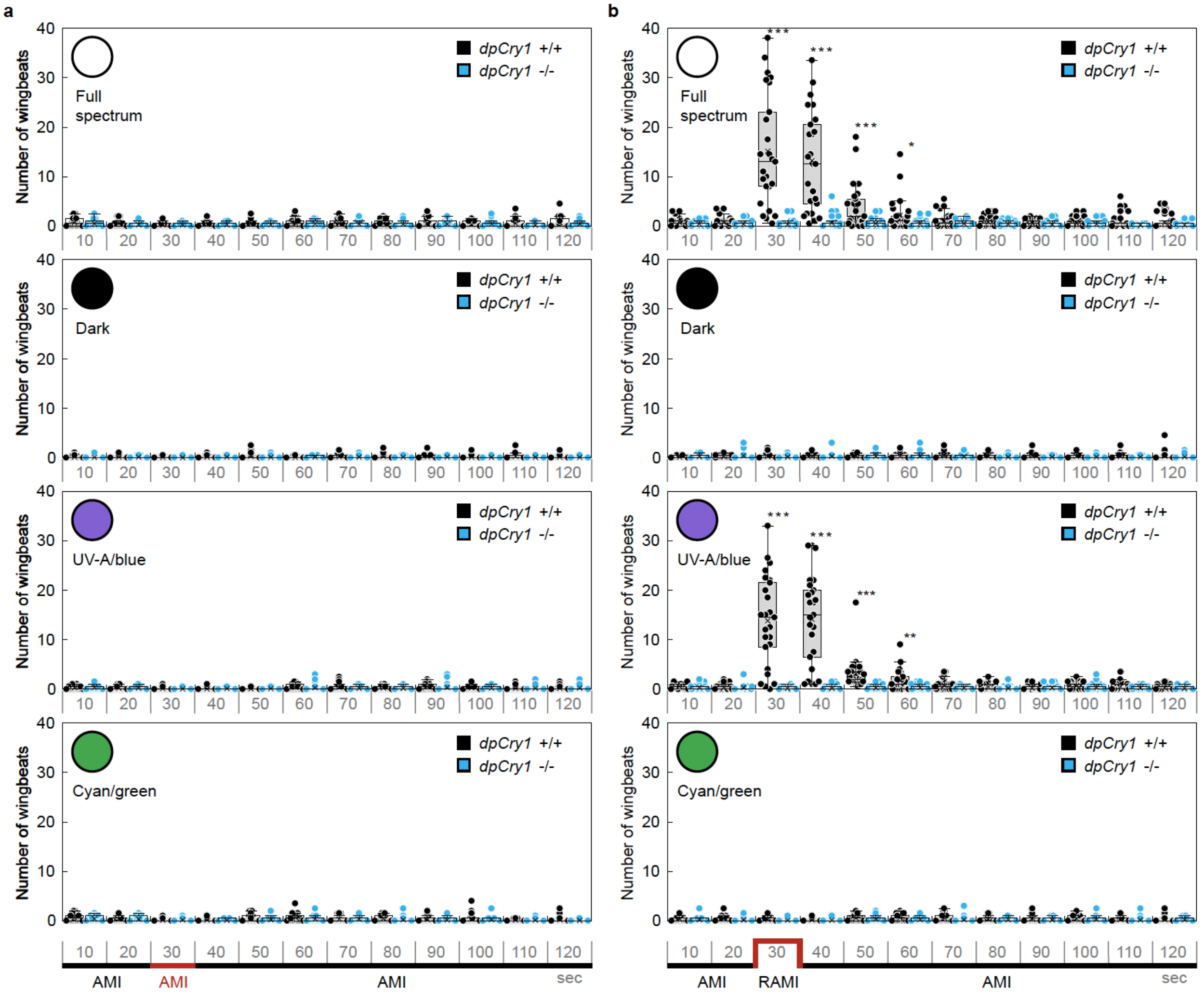

Supplementary Figure 3: Magnetic responses of *dpCry1*$^{+/+}$ (black) and *dpCry1*$^{-/-}$ (blue) monarchs tested blindly to genotypes and using a double-wrapped coil system to a control ambient magnetic inclination (AMI) (a) or a reversal of magnetic inclination (RAMI) (b) under different lighting conditions. Antiparallel and parallel currents were injected in the double-wrapped coils to generate control AMI and RAMI conditions, respectively. Each box plot shows the number of wingbeats for every 10 seconds (sec) time bin (median, center line; interquartile range (IQR), box; 1.5 × IQR, whiskers). Each dot represents an average value for the response of an individual measured in two independent tests (n = 25 and 23 for *dpCry1*$^{+/+}$ and *dpCry1*$^{-/-}$, respectively). In each lighting condition (full spectrum, white circle; darkness, black circle; UV-A/blue, violet circle; cyan/green, green circle), statistical significance between genotypes was tested for each time bin using a two-tailed Mann-Whitney U test at $p < 0.05$ (Full spectrum in **b**: * $p = 0.013$ for 6th 10 sec time bin, *** $p = 4.639E\text{-}9$, $p = 2.915E\text{-}8$ and $p = 1.180E\text{-}6$ for 3rd, 4th, and 5th 10 sec time bins respectively; UV-A/blue in **b**: ** $p = 0.004$ for 6th 10 sec time bin, *** $p = 1.201E\text{-}8$, $p = 2.558E\text{-}9$, and $p = 7.901E\text{-}9$ for 3rd, 4th, and 5th 10 sec time bins respectively).

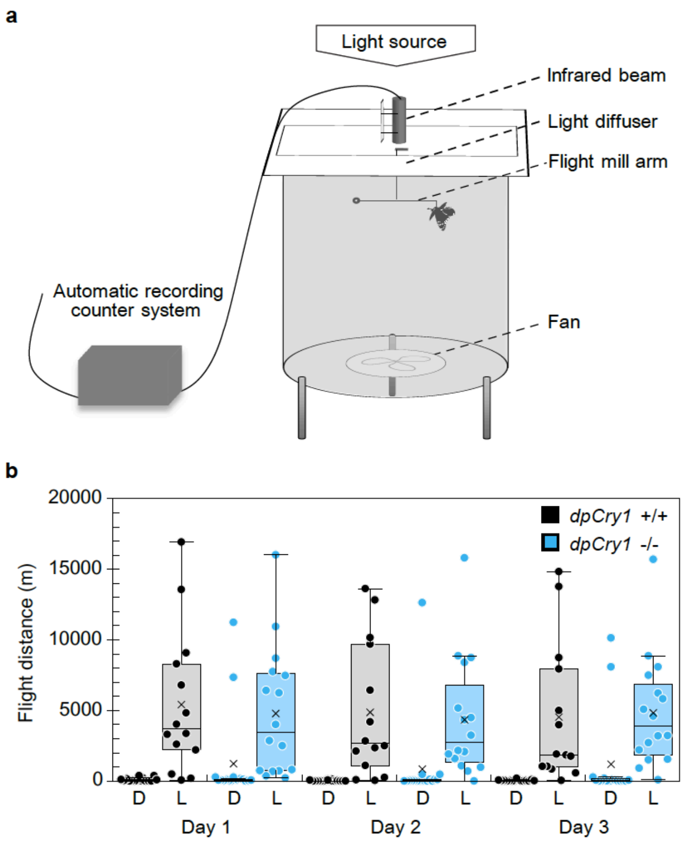

Supplementary Figure 4: *DpCry1*$^{+/+}$ and *dpCry1*$^{-/-}$ monarchs flight activity. a, Schematic of the flight mill in which a monarch is suspended to one end of a balanced flight mill arm. The number of revolutions of the arm is automatically recorded using an infrared beam connected to an automatic recording counter system. **b**, Distance flown in meters (m) by *dpCry1*$^{+/+}$ (black) and *dpCry1*$^{-/-}$ (blue) monarchs over three days under a 15-hours light: 9-hours dark cycle at 21°C. Each box plot shows the flight distance under light (L) or dark (D) conditions each day (median, center line; interquartile range (IQR), box; 1.5 × IQR, whiskers). Each dot represents the distance flown by an individual monarch (n = 14 and 16 for *dpCry1*$^{+/+}$ and *dpCry1*$^{-/-}$, respectively). Statistical significance between genotypes was tested for each lighting condition using a two-tailed Mann-Whitney U test at $p < 0.05$. No significance was found.

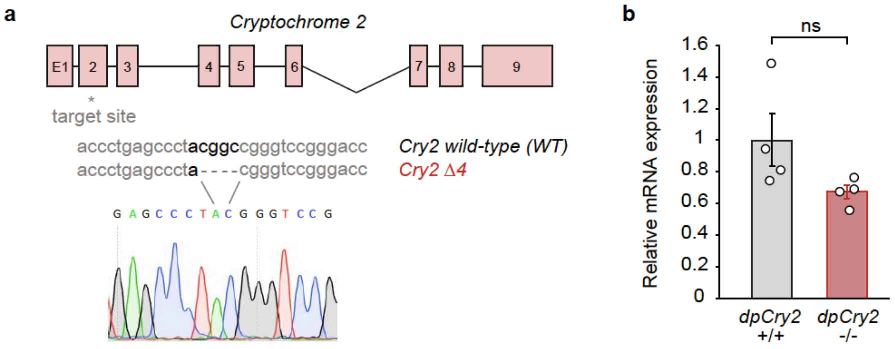

c

```
CRY2  WT    MSVAETLPLRARSPTAQKSSQPAGVPKEKHTVHWFRKGLRLHDNPALREGLVDATTFRCV   60
CRY2  Δ4    MSVAETLPLRARSPTAQKSSQPAGVPKEKHTVHWFRKGLRLHDNPALREGLVDATTFRCV   60

CRY2  WT    FIIDPWFASSSNVGINKWRFLLQCLEDLDKNLRKLNSRLFVVRGQPADALPKLFREWGTT   120
CRY2  Δ4    FIIDPWFASSSNVGINKWRFLLQCLEDLDKNLRKLNSRLFVVRGQPADALPKLFREWGTT   120

CRY2  WT    ALTFEEDPEPYGRVRDHNIMTKCREVGIQVTSRVSHTLYKLDDIIEKNGGKAPLTYHQFQ   180
CRY2  Δ4    ALTFEEDPEPYGSGTIIL*-----------------------------------------   138

CRY2  WT    ALIASMPPPPSAEPTISLETLNRAVTPISDNHDERFGVPTLEELGFDTEGLKPPIWIGGE   240
CRY2  Δ4    ------------------------------------------------------------   138

CRY2  WT    NEALLRLERHLERKAWVASFGRPKMTPESLLSSQTGLSPYLRFGCLSTRLFYYQLSELYK   300
CRY2  Δ4    ------------------------------------------------------------   138
                                                        Trp3
CRY2  WT    RIKQERPPLSLHGQILWREFFYCAATRNPNFDRMEGNPICVQIPWEKNQEALKKWANGQT   360
CRY2  Δ4    ------------------------------------------------------------   138
                                                Trp4 Trp2
CRY2  WT    GFPWIDAIMIQLRNDGWIHHLARHAVACFLTRGDLWISWEEGMKVFDELLLDADWSVNAG   420
CRY2  Δ4    ------------------------------------------------------------   138
             Trp1
CRY2  WT    MWMWLSCSSFFQQFFHCYCPVRFGRKTDPNGDFIRKYIPVLKNMPTRYIHEPWVCPEEIQ   480
CRY2  Δ4    ------------------------------------------------------------   138

CRY2  WT    KSIRCIIGKDYPMPIVDHTKASEINLERIKQVYAQLAKFKPQGALIPQMLQRPNVLQSSP   540
CRY2  Δ4    ------------------------------------------------------------   138

CRY2  WT    SPTSIIANINQSNYLCSQSSDVPTPTNQTTNQFKEDAVFLKPTVNNIKSNVDKQQQFKQV   600
CRY2  Δ4    ------------------------------------------------------------   138

CRY2  WT    VIVQEDKHSENQRHSVGNKYIVNEINKNINDIPVKQNNYDFKALTLNLNKFSNEPLTFLN   660
CRY2  Δ4    ------------------------------------------------------------   138

CRY2  WT    QTPNKNESFGQDVNNVIDVYSTSKPKFYFTDNGVITHNENAQTFKRDSYSDNYNKESTGS   720
CRY2  Δ4    ------------------------------------------------------------   138

CRY2  WT    NRVGEVHSNNPQTDKISSEKKN                                         742
CRY2  Δ4    ----------------------                                         138
```

Supplementary Figure 5: *Cryptochrome 2* loss-of-function monarch mutant. a, Schematic representation of monarch *dpCry2* genomic locus, with the site previously targeted to generate a 4-bp deletion (Δ4) and establish the *dpCry2* loss-of-function mutant line used in this study[1]. **b,** Relative *dpCry2* mRNA expression in brains of wild-type ($^{+/+}$; gray) and homozygous *dpCry2 Δ4* mutant ($^{-/-}$; red) monarchs (n = 4 for each genotype). Data are presented as mean values ± SEM. Unpaired Student's t test: $p = 0.1$; ns, non significant. **c,** Amino acid sequence alignment of wild-type (WT) and truncated loss-of-function (Δ4) dpCRY2 proteins. The four tryptophan (Trp) residues involved in the Trp tetrad are highlighted in dark blue.

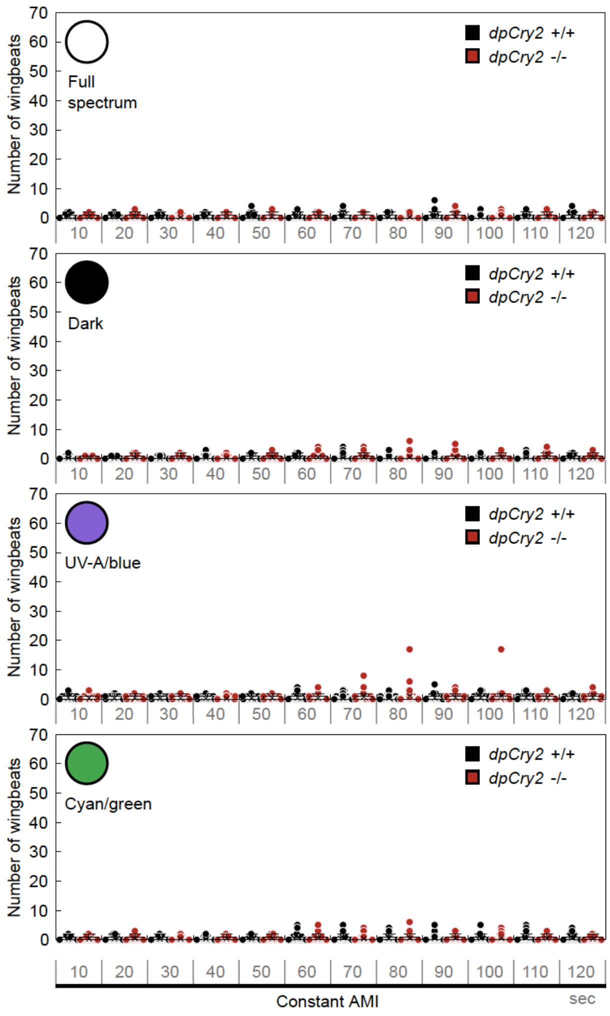

Supplementary Figure 6: Absence of magnetic response in *dpCry2*$^{+/+}$ (black) and *dpCry2*$^{-/-}$ (red) monarchs subjected to control constant ambient magnetic inclination (AMI) under different lighting conditions. Each box plot shows the number of wingbeats for every 10 seconds (sec) time bin (median, center line; interquartile range (IQR), box; 1.5 × IQR, whiskers). Each dot represents the response of an individual (n = 30 for *dpCry2*$^{+/+}$ and n = 25 for *dpCry2*$^{-/-}$). In each lighting condition (full spectrum, white circle; darkness, black circle; UV-A/blue, violet circle; cyan/green, green circle), statistical significance between genotypes was tested for each time bin using a two-tailed Mann-Whitney U test at $p < 0.05$.

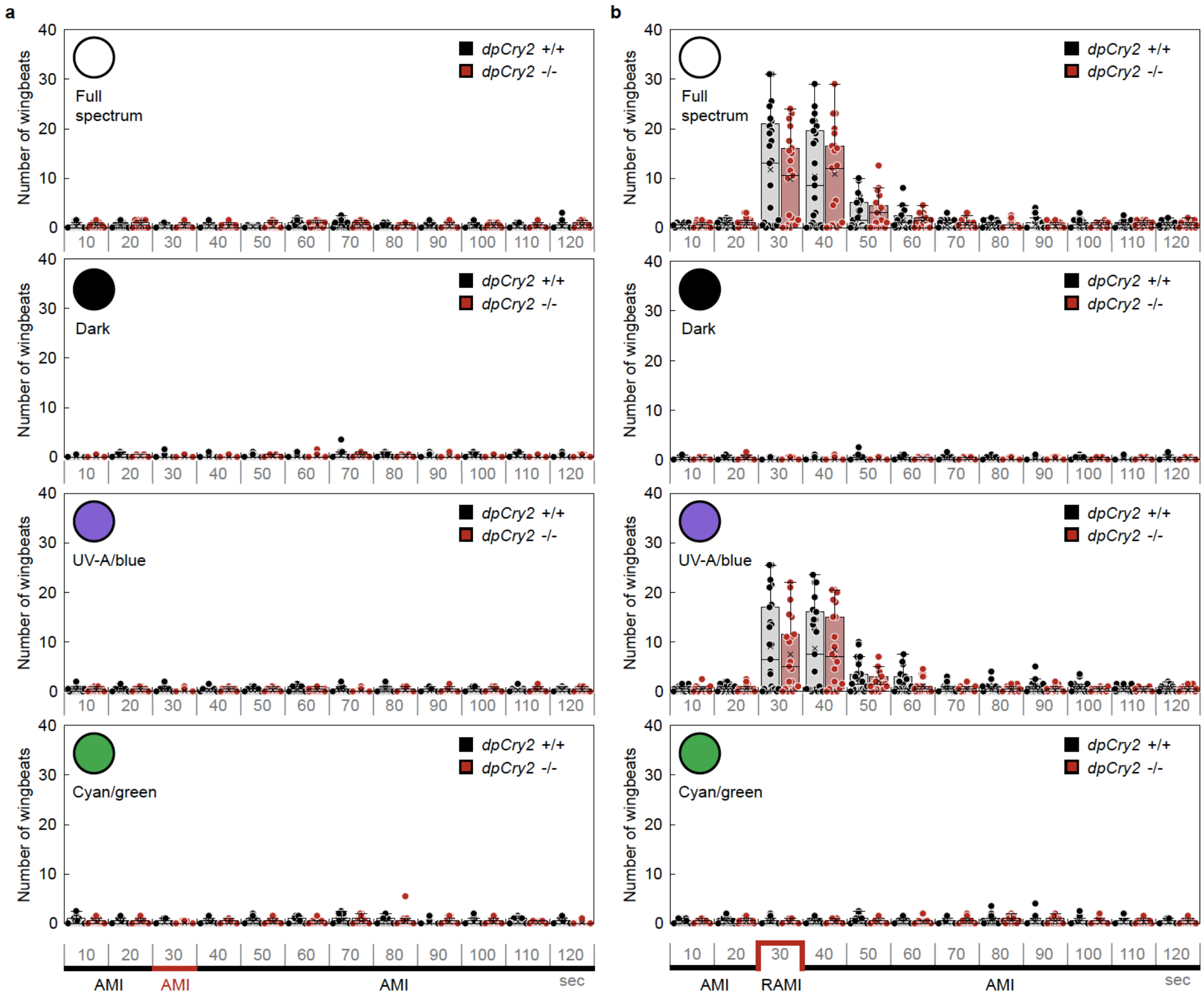

Supplementary Figure 7: Magnetic responses of *dpCry2*$^{+/+}$ (black) and *dpCry2*$^{-/-}$ (red) monarchs tested blindly to genotypes and using a double-wrapped coil system to a control ambient magnetic inclination (AMI) (a) or a reversal of magnetic inclination (RAMI) (b) under different lighting conditions. Antiparallel and parallel currents were injected in the double-wrapped coils to generate control AMI and RAMI conditions, respectively. Each box plot shows the number of wingbeats for every 10 seconds (sec) time bin (median, center line; interquartile range (IQR), box; 1.5 × IQR, whiskers). Each dot represents an average value for the response of an individual measured in two independent tests (n = 25 and 21 for *dpCry2*$^{+/+}$ and *dpCry2*$^{-/-}$, respectively). In each lighting condition (full spectrum, white circle; darkness, black circle; UV-A/blue, violet circle; cyan/green, green circle), statistical significance between genotypes was tested for each time bin using a two-tailed Mann-Whitney U test at $p < 0.05$. No significance was found.

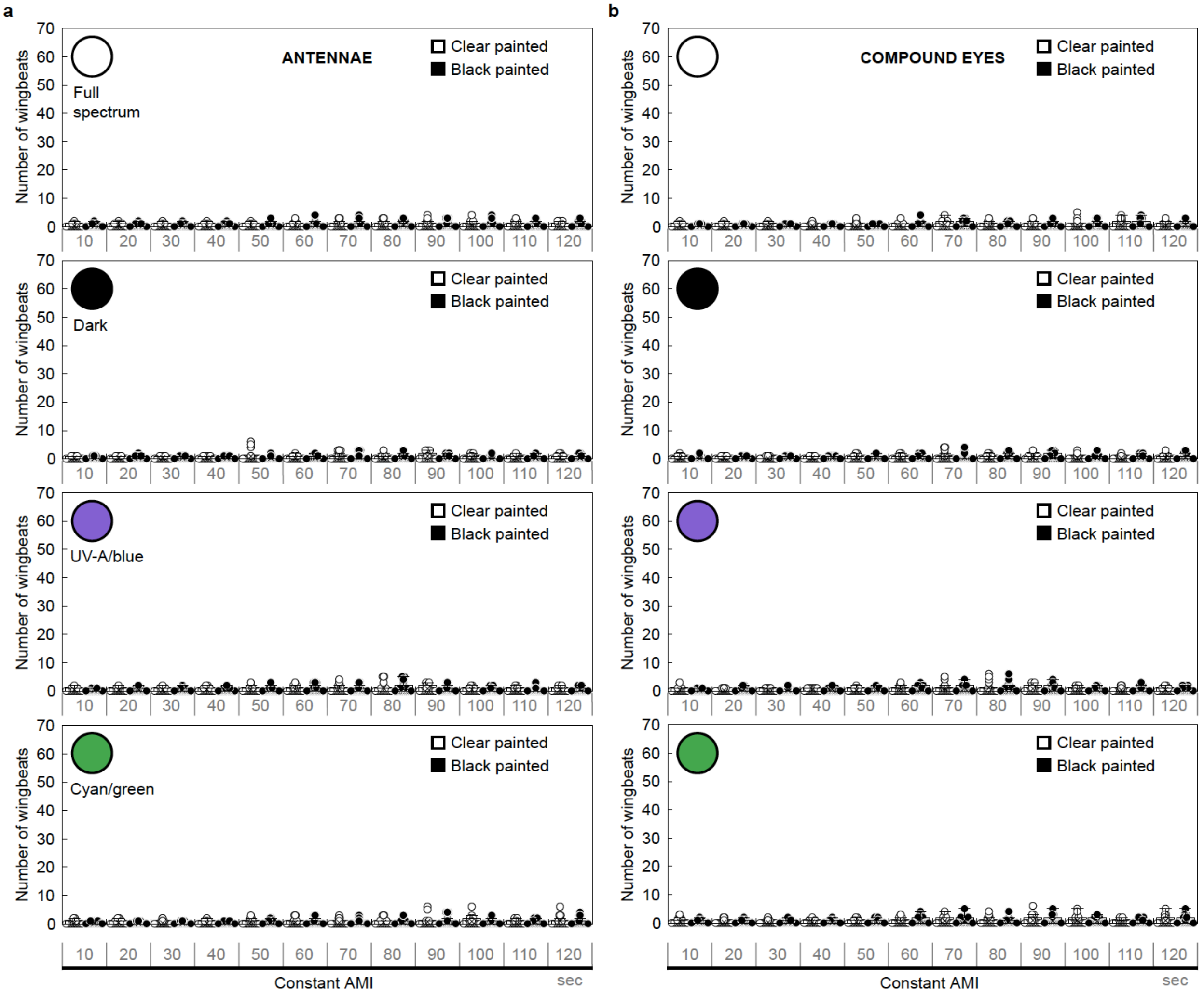

Supplementary Figure 8: Absence of magnetic response in monarchs with black painted (black) and clear painted (white) antennae (a) or compound eyes (b) and subjected to control constant ambient magnetic inclination (AMI) under different lighting conditions. Each box plot shows the number of wingbeats for every 10 seconds (sec) time bin (median, center line; interquartile range (IQR), box; 1.5 × IQR, whiskers). Each dot represents the response of an individual (n = 18 for each painting group). In each lighting condition (full spectrum, white circle; darkness, black circle; UV-A/blue, violet circle; cyan/green, green circle), statistical significance between painting groups was tested for each time bin using a two-tailed Mann-Whitney U test at $p < 0.05$.

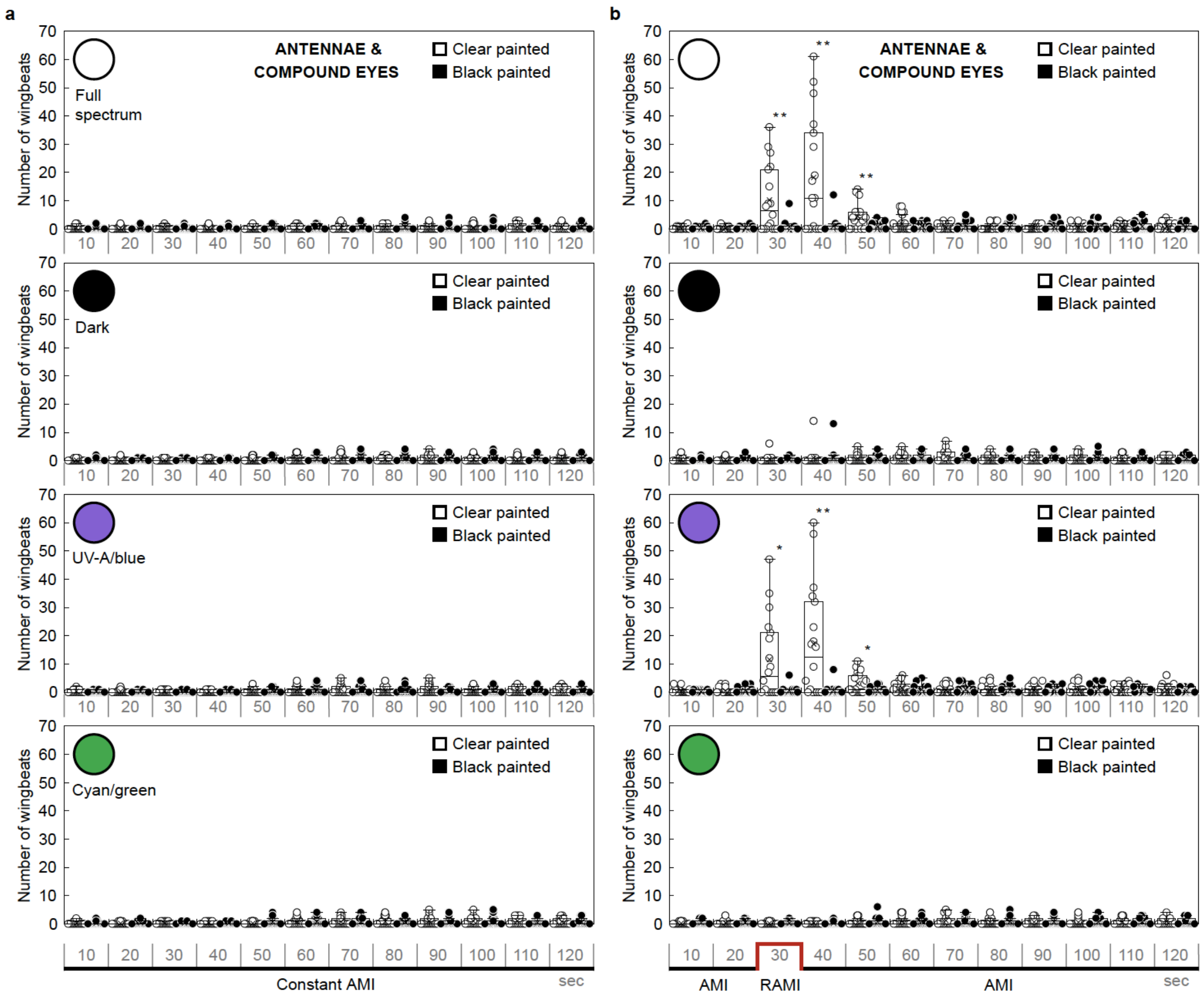

Supplementary Figure 9: Blocking the light input to both the compound eyes and the antennae impairs magnetic responses of monarchs to a reversal of ambient magnetic inclination (RAMI). a, Absence of magnetic response in monarchs with antennae and compound eyes painted black (black) or painted clear (white) and subjected to control constant AMI under different lighting conditions. **b,** Magnetic responses in monarchs with antennae and compound eyes painted black and painted clear and subjected to a RAMI under different lighting conditions. Each box plot shows the number of wingbeats for every 10 seconds (sec) time bin (median, center line; interquartile range (IQR), box; 1.5 × IQR, whiskers). Each dot represents the response of an individual (n = 18 for each painting group). In each lighting condition (full spectrum, white circle; darkness, black circle; UV-A/blue, violet circle; cyan/green, green circle), statistical significance between painting groups was tested for each time bin using a two-tailed Mann-Whitney U test at $p < 0.05$ (Full spectrum in **b**: ** $p = 0.009$, $p = 0.005$ and $p = 0.003$ for 3rd, 4th and 5th 10 sec time bins respectively; UV-A/blue in **b**: * $p = 0.013$ and $p = 0.047$ for 3rd and 5th 10 sec time bins respectively, ** $p = 0.004$ for 4th 10 sec time bin).

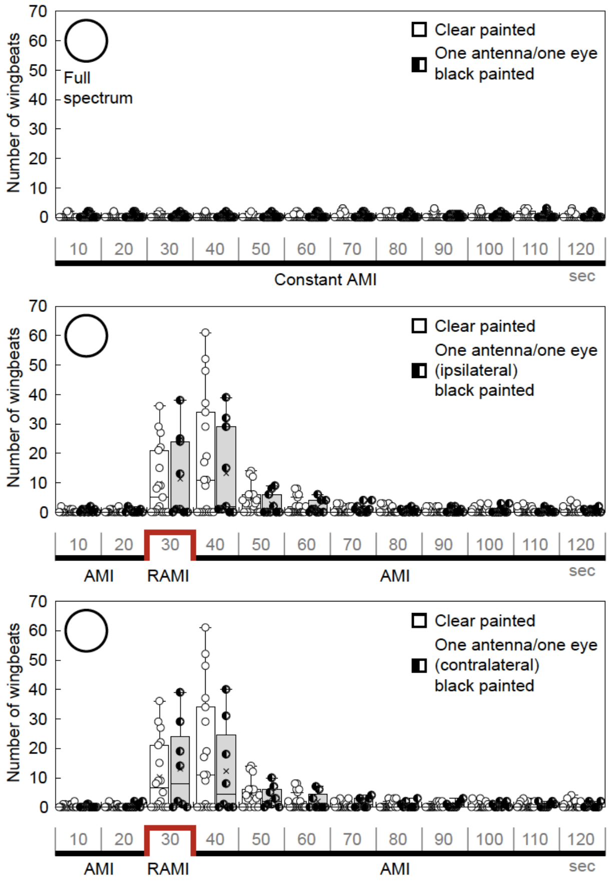

Supplementary Figure 10: One antenna and one compound eye are sufficient for monarch light-dependent magnetoreception, irrespective of whether they are ipsilateral or contralateral. Monarchs with both antennae and both eyes painted clear (white bars and dots) were used as controls (n = 18). Only one antenna and one eye were painted with black paint (black bars and black and white dots), either ipsilaterally (n = 9) or contralaterally (n = 9). **Top,** Absence of magnetic response in monarchs subjected to control constant ambient magnetic inclination (AMI) under full-spectrum light (white circle). **Middle,** Magnetic responses of monarchs with one antenna and the ipsilateral eye painted back and subjected to a RAMI (red line) under full-spectrum light. **Bottom,** Magnetic responses of monarchs with one antenna and the contralateral eye painted back and subjected to a RAMI under full-spectrum light. Control monarchs painted clear were replotted in middle and bottom panels. Each box plot shows the number of wingbeats for every 10-s time bin (median, center line; interquartile range (IQR), box; 1.5 × IQR, whiskers). Each dot represents the response of an individual. Statistical significance between painting groups was tested for each time bin using a two-tailed Mann-Whitney U test at $p < 0.05$.

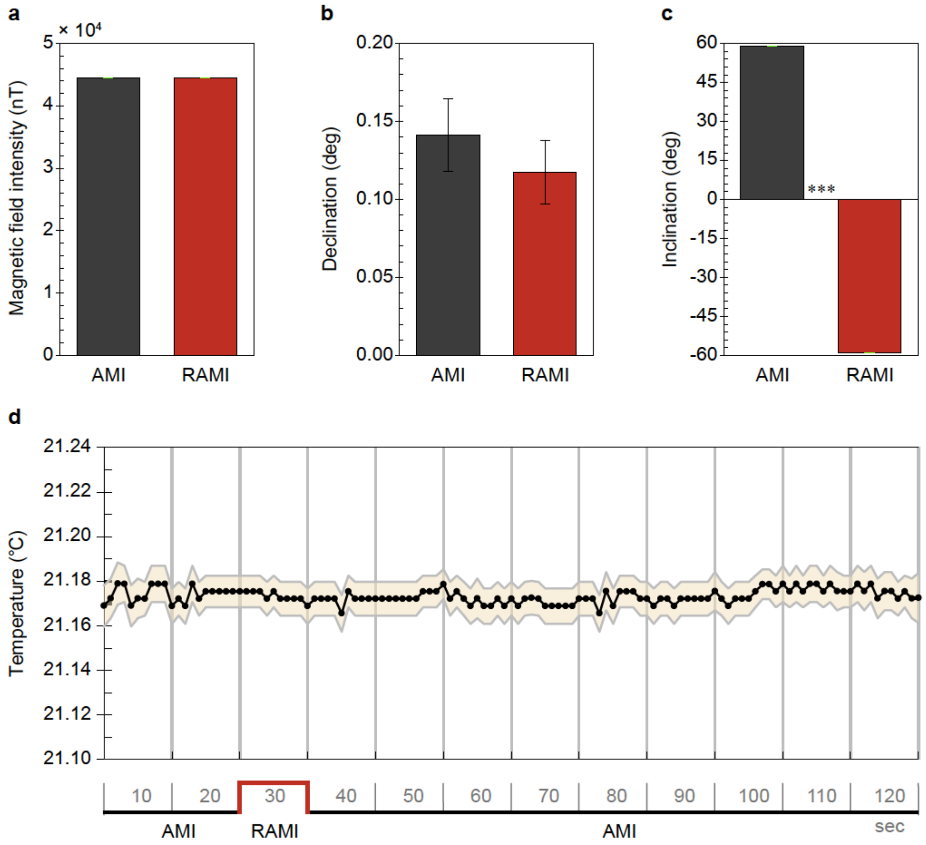

Supplementary Figure 11: Measurements of magnetic field parameters used in the behavioral assay and heat generated by the coils system. The magnetic field intensity (**a**), magnetic declination (**b**) and magnetic inclination (**c**) were measured and recorded for 120 seconds under control AMI (dark gray) and RAMI (red) conditions prior to testing monarch responses (n = 46). Statistical significance for each magnetic field parameter between AMI and RAMI was tested using a two-tailed Mann-Whitney U test at $p < 0.05$ ($^{***}p = 0.0E0$). Data are presented as mean values ± SEM. **d,** The temperature generated by the coil system before and after a RAMI treatment was measured every second over 120 seconds (sec) at the level at which monarchs are suspended inside the apparatus (n = 30). The black line with dots represents mean values and the yellow band represents SEM. No obvious changes in temperature were found before, during, or after the RAMI.

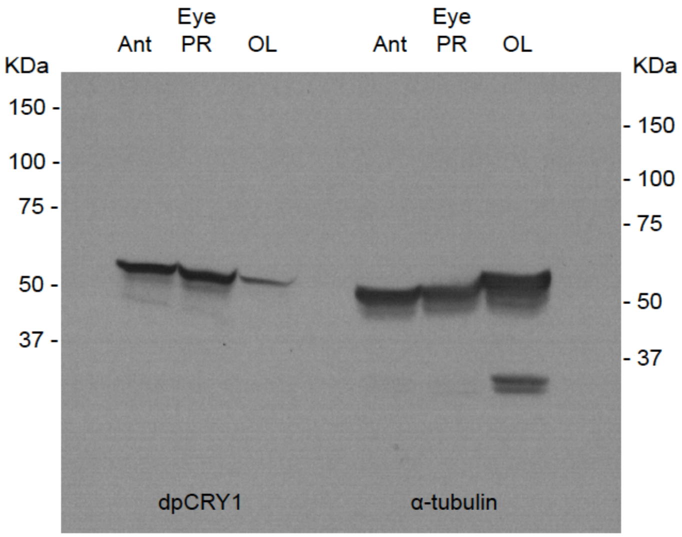

Supplementary Figure 12: Uncropped western blot. Full scan image of the western blot shown in Figure 4e.

Supplementary Table

Supplementary Table 1. Oligonucleotides used in this study.

gRNAOligoCry1_F	5’-TAGGGACCTGAACGGCGTCAACTT-3’
gRNAOligoCry1_R	5’-AAACAAGTTGACGCCGTTCAGGTC-3’
sgRNACry1_F	5’-ATTGAGCCTCAGGAAACAGC-3’
sgRNACry1_R	5’-AAAAGCACCGACTCGGTGCC-3’
gDNACry1_F	5’-CTGGCCTTGATCGCTTACAG-3’
gDNACry1_R	5’-CGTACTCCACAGCCAATCTC-3’
cDNACry1_F	5’-CGAGCACGTCGCACACA-3’
cDNACry1_R	5’-TCCTCCATTGGCCTTGATGA-3’
cDNArp49_F	5’-TGCGCAGGCGTTTTAAGG-3’
cDNArp49_R	5’-TTGTTTGATCCGTAACCAATGC-3’
pDNACry1_F	5’-CTCGAGTCTAGACTTGGTGGTAATGTCATTTG-3’
pDNACry1 Δ2_R	5’-GAACGGCGTCCTTCGGATCGCTGCCTGAG-3’
pDNACry1 Δ2_F	5’-GATCCGAAGGACGCCGTTCAGGTCG-3’
pDNACry1_R	5’-AAACCCTCTAGATCATGTGGTGGTGAGCTCAG-3’

Supplementary Methods

Assay for magnetic responses of *dpCry1* and *dpCry2* mutants and wild-type siblings tested blindly using a double wrapped coils system

A custom-build double wrapped 3-axis Helmholtz coils system of the exact same dimensions as the single wrapped coil system was used to test magnetic responses of *dpCry1* and *dpCry2* homozygous mutants and wild-type siblings under different lighting conditions following the protocol described in the main text, with some modifications. To shield butterflies from potential environmental electromagnetic noise[2], the double wrapped coils system was placed inside a six-sided Faraday cage (183 cm × 183 cm × 245 cm) which was grounded during the experiment, and the camera used to record behavioral activity was shielded with a copper mesh. The ambient magnetic inclination and its reversal were generated within the double-wrapped coils system by injecting the same amount of current through both coils either in antiparallel (AMI control) or parallel (RAMI treatment) directions to control for any possible artifacts such as vibrations or heat generated while charging the coils[3]. The experimenter was also blind to the monarch genotype in both behavioral tests and data analyses.

Flight activity assay

The general activity levels of *dpCry1*$^{+/+}$ and *dpCry1*$^{-/-}$ monarchs were quantitated using a custom built flight mill inside a plastic barrel. Inside the barrel, individual monarchs were suspended by a tether glued to their thorax to one end of a flight arm positioned on a horizontal plane and counterbalanced on the opposite end by a weight (see Supplementary Fig. 4a). Once the butterfly flies actively, it moves in a circular horizontal plane. An infrared beam placed on top of the barrel in the path of the arm and connected to an automatic counter system automatically recorded the number of revolutions made during active flight. A fan was built-in at the bottom of the barrel, but was not turned on during the tests to avoid any disturbance from the airflow. *DpCry1* $^{-/-}$ and *dpCry1*$^{+/+}$ were tested blindly to genotype under a 15-hours light (L): 9-hours dark (D) cycle at 21°C. All monarchs were acclimated for one day before the three days of locomotor activity test and were fed daily with 25% honey solution ~15 minutes before the light off. The

distance flown by each individual was extrapolated based on the number of revolutions made within a given time period and the radius of the circular flight path (*i.e.*, half the length of the flight arm). Data were collected using a commercial data acquisition software (CHMBDD, MB-96).

Real-time qPCR

Adult *dpCry1* and *dpCry2* homozygous mutant monarchs and their wild-type siblings were entrained seven days after eclosion in 15-hours light: 9-hours dark cycles at 25°C. Brains were dissected in 0.5X RNA later (Invitrogen) at each gene's respective peak of expression in wild-type monarchs (ZT8 for *dpCry1* and ZT20 for *dpCry2*). Dissections at ZT20 were performed under red light. Total RNA was extracted using a RNeasy Mini kit (Qiagen) and reverse-transcribed with SuperScript II Reverse Transcriptase (Thermo Scientific) and random hexamers, following the manufacturers' instructions. Quantifications of gene expression were performed on a QuantStudio 6 Flex Real-Time PCR System (Thermo Scientific) using iTaq Universal SYBR Green Supermix (Bio-Rad), as described in methods in the main text. Primers were as follows (Supplementary Table 1): *cDNACry1_F*, 5'-CGAGCACGTCGCACACA-3'; *cDNACry1_R*, 5'-TCCTCCATTGGCCTTGATGA-3'; *cDNACry2_F*, 5'-TGGCTCTCATGCTCGTCTTTC -3'; *cDNACry2_R*, 5'-ACCGCACTGGACAGTAGCAAT -3'; *cDNArp49_F*, 5'-TGCGCAGGCGTTTTAAGG-3'; *cDNArp49_R*, 5'-TTGTTTGATCCGTAACCAATGC-3'.

DpN1 transfection and western blotting of overexpressed FLAG-dpCRY1 and FLAG-dpCRY1 Δ2

DpN1 cells were cultured in Grace's insect medium (Gibco) supplemented with 10% fetal bovine serum (Seradigm/VWR). The cells were maintained at 28°C in 25-cm^2 plug seal flasks and split every 4 days. The DpN1 expression plasmids pBA_FLAG_*dpCry1* and pBA_FLAG_*dpPer* used were reported previously[4]. The expression plasmid pBA_FLAG_*dpCry1 Δ2* was generated by subcloning a mutated *dpCry1* bearing the same 2 bp deletion than that of the *dpCry1* mutant monarch line into the pBA_FLAG

vector[4] using the *XbaI* site of the multiple cloning site. The *dpCry1 Δ2* fragment was generated by overlapping PCR of two fragments respectively amplified from the pBA_FLAG_*dpCry1* plasmid with the following primers: *dpCry1pDNACry1_F*, 5'-CTCGAGTCTAGACTTGGTGGTAATGTCATTTG-3' and *pDNACry1 Δ2_R*, 5'-GAACGGCGTCCTTCGGATCGCTGCCTGAG-3', and *pDNACry1 Δ2_F*, 5'-GATCCGAAGGACGCCGTTCAGGTCG-3' and *pDNACry1_R*, 5'-AAACCCTCTAGATCATGTGGTGGTGAGCTCAG-3'.

DpN1 cells were co-transfected with 100 ng/well of pBA-FLAG-*dpPer* and either 100 ng/well of pBA-FLAG-*dpCry1* or pBA-FLAG-*dpCry1 Δ2*. Cells were split into 6-well dishes and incubated at 28°C for 1 day so that cultures were ~80% confluent. The cells were then incubated in 600 µl serum-free Grace medium (Invitrogen) premixed with plasmids and 5 µl/well Cellfectin (Invitrogen) for 5 hours, and Grace's medium supplemented with 10% fetal bovine serum (1400 µl) was added at the end of transfection. The cells were then incubated for 2 days before harvesting for Western blot analysis. For western blot, cells were washed with ice-cold PBS and lysed with 50 µl 1X Passive Lysis buffer (Promega). For each sample, 20 µl of heated and DTT-denatured protein were loaded and separated onto a 7.5% SDS PAGE. FLAG-dpPER, FLAG-dpCRY1 and FLAG-dpCRY1 Δ2 were detected using a mouse anti-FLAG primary antibody (1:5000; Sigma F3165) and a goat anti-mouse IgG-HRP secondary antibody (1:10000; Biorad 170-6516).

References

1 Merlin, C., Beaver, L. E., Taylor, O. R., Wolfe, S. A. & Reppert, S. M. Efficient targeted mutagenesis in the monarch butterfly using zinc-finger nucleases. *Genome Res* **23**, 159-168 (2013).

2 Engels, S. *et al.* Anthropogenic electromagnetic noise disrupts magnetic compass orientation in a migratory bird. *Nature* **509**, 353-356 (2014).

3 Kirschvink, J. L., Winklhofer, M. & Walker, M. M. Biophysics of magnetic orientation: strengthening the interface between theory and experimental design *J R Soc Interface* **7** **Suppl 2**, S179-191 (2010).

4 Zhu, H. *et al.* Cryptochromes define a novel circadian clock mechanism in monarch butterflies that may underlie sun compass navigation. *PLoS Biol* **6**, e4 (2008).
