# Supplementary material for: Cryptochrome 1 mediates light-dependent inclination magnetosensing in monarch butterflies
Source: Nat Commun. 2021 Feb 3;12:771. doi: 10.1038/s41467-021-21002-z (PMC7859408; doi:10.1038/s41467-021-21002-z)
Supplement: Supplementary file 3 — Reporting Summary [file 41467_2021_21002_MOESM3_ESM.pdf]

nature research

Corresponding author(s): Christine Merlin; Guijun Wan

Last updated by author(s): Dec 20, 2020

Reporting Summary

Nature Research wishes to improve the reproducibility of the work that we publish. This form provides structure for consistency and transparency in reporting. For further information on Nature Research policies, see Authors & Referees and the Editorial Policy Checklist .

Statistics

For all statistical analyses, confirm that the following items are present in the figure legend, table legend, main text, or Methods section.

n/a	Confirmed	
☐	☒	The exact sample size (*n*) for each experimental group/condition, given as a discrete number and unit of measurement
☐	☒	A statement on whether measurements were taken from distinct samples or whether the same sample was measured repeatedly
☐	☒	The statistical test(s) used AND whether they are one- or two-sided<br>*Only common tests should be described solely by name; describe more complex techniques in the Methods section.*
☒	☐	A description of all covariates tested
☐	☒	A description of any assumptions or corrections, such as tests of normality and adjustment for multiple comparisons
☐	☒	A full description of the statistical parameters including central tendency (e.g. means) or other basic estimates (e.g. regression coefficient) AND variation (e.g. standard deviation) or associated estimates of uncertainty (e.g. confidence intervals)
☐	☒	For null hypothesis testing, the test statistic (e.g. *F*, *t*, *r*) with confidence intervals, effect sizes, degrees of freedom and *P* value noted<br>*Give P values as exact values whenever suitable.*
☒	☐	For Bayesian analysis, information on the choice of priors and Markov chain Monte Carlo settings
☒	☐	For hierarchical and complex designs, identification of the appropriate level for tests and full reporting of outcomes
☒	☐	Estimates of effect sizes (e.g. Cohen's *d*, Pearson's *r*), indicating how they were calculated

Our web collection on statistics for biologists contains articles on many of the points above.

Software and code

Policy information about availability of computer code

Data collection	A commercial data acquisition software (CHMBDD, MB-96) was used to collect locomotor activity data.
Data analysis	IBM SPSS Statistics 25 was used for our statistical analyses.

For manuscripts utilizing custom algorithms or software that are central to the research but not yet described in published literature, software must be made available to editors/reviewers. We strongly encourage code deposition in a community repository (e.g. GitHub). See the Nature Research guidelines for submitting code & software for further information.

Data

Policy information about availability of data

All manuscripts must include a data availability statement. This statement should provide the following information, where applicable:

- Accession codes, unique identifiers, or web links for publicly available datasets
- A list of figures that have associated raw data
- A description of any restrictions on data availability

Source data are provided with this paper. A reporting summary for this Article is available as a Supplementary Information file.

Field-specific reporting

Please select the one below that is the best fit for your research. If you are not sure, read the appropriate sections before making your selection.

☒ Life sciences ☐ Behavioural & social sciences ☐ Ecological, evolutionary & environmental sciences

For a reference copy of the document with all sections, see nature.com/documents/nr-reporting-summary-flat.pdf

Life sciences study design

All studies must disclose on these points even when the disclosure is negative.

Sample size	No sample-size calculation was performed for either behavioral or qPCR data. For magnetic hyperactivity behavior experiments, our sample size ranged from 18 to 32 individual butterflies. For locomotor activity behavior experiments, our sample size ranged from 14 to 16 individual butterflies. The absolute number chosen depended on availability in the laboratory at the time of the experiments, and were arbitrary. Because the responses observed are quite robust, we believe that the sample-size chosen is large enough. For qPCR data, we chose a sample-size of up to 6 to be above the minimal number of 3 replicates for statistical analyses.
Data exclusions	For magnetic hyperactivity behavior experiments, monarchs being overactive (flapping wings without stopping and without stimulation) or underactive (not reacting to a mechanical stimulation) were excluded from data collection (i.e., their exclusion was pre-established). This exclusion was done because we needed monarchs to be calm at the start of the experiment but that also had the potential to respond to stimulation. No monarchs tested behaviorally was later removed from the dataset and all were reported on the figures (see individual dots).
Replication	For behavioral data, reproducibility was assessed by testing every individual again one to two more times on different days, and verify that each monarch was presenting a similar response at least one time. Replication was successful in the majority of cases and raw data are presented in a Source Data file. For each butterfly tested in a non-blind fashion (main figures), only quantifications (number of wingbeats) of the first of the 2 or 3 experimental tests are reported to minimize the effect of aging (as the ones tested three times were older). For each butterfly tested blindly with respect to genotype (supplementary figures), quantifications (number of wingbeats) of two experimental tests are reported as averages.
Randomization	For butterflies tested in a non-blind fashion, samples were allocated into experimental groups based on their genotypes defined by PCR-based assays or painting status prior to the experiment. Each day of testing, both Cry1-/- and Cry2-/- mutant monarchs were tested in a random order, but each mutant was always tested in pair with a wild-type sibling. Similar protocols were used for the painted monarchs, were black painted individuals were tested in pair with clear painted controls. The experiment was designed in this way to minimize possible differences in the behavioral responses due to day-to-day variations in humidity, air pressure, and other unknown variables that may affect behavior.<br>For butterflies tested blindly with respect to the genotype, monarchs were tested randomly.
Blinding	In a first set of experiments (presented in main figures), investigators were not blinded to group allocation during data collection and analysis. Blinding was not applied because the magnetic response in this work are quantitative and recorded using an infrared LED beam, which precludes unconscious bias by the experimenter.<br>In a second set of experiments requested by the reviewers (presented in supplementary figures), investigators were blinded to genotype during data collection and analysis.

Reporting for specific materials, systems and methods

We require information from authors about some types of materials, experimental systems and methods used in many studies. Here, indicate whether each material, system or method listed is relevant to your study. If you are not sure if a list item applies to your research, read the appropriate section before selecting a response.

Materials & experimental systems

n/a	Involved in the study	
☐	☒	Antibodies
☐	☒	Eukaryotic cell lines
☒	☐	Palaeontology
☐	☒	Animals and other organisms
☒	☐	Human research participants
☒	☐	Clinical data

Methods

n/a	Involved in the study	
☒	☐	ChIP-seq
☒	☐	Flow cytometry
☒	☐	MRI-based neuroimaging

Antibodies

Antibodies used	Anti monarch butterfly CRYPTOCHROME 1 primary antibody raised in guinea pig (dpCRY1-GP37).<br>Mouse anti-alpha tubulin monoclonal primary antibody (Sigma B-5-1-2).<br>Peroxidase affinipure donkey anti-guinea pig IgG secondary antibody (Jackson ImmunoResearch #706-035-148).<br>Goat anti-mouse IgG HRP secondary antibody (Invitrogen 31430).<br>Mouse anti-FLAG monoclonal primary antibody (Sigma F3165)<br>Goat anti-mouse IgG-HRP secondary antibody (Biorad 170-6516).
Validation	The specificity of the anti monarch butterfly CRYPTOCHROME 1 primary antibody used in this study (dpCRY1-GP37) was previously validated by western-blot of monarch CRY1, Antheraea pernyi CRY1 (another lepidoptera), and Drosophila CRY expressed in Drosophila Schneider's S2 cells (in Figure S6 of: Sauman, I., Briscoe, A.D., Zhu, H., Shi, D., Froy, O., Stalleichen, J., Yuan, Q., Casselman, A., Reppert, S.M. (2005). Connecting the navigational clock to sun compass input in monarch butterfly brain. Neuron 46:457-467).<br>The commercially available mouse anti-alpha tubulin monoclonal primary antibody (Sigma B-5-1-2) used to probe tubulin in

monarch tissues (antennae, eye photoreceptors and optic lobes) has been validated for western-blot with reactivity in a variety of species, as documented on the manufacturer's website. Cross-reactivity with monarch tubulin is confirmed in our data by showing signal at the expected molecular weight of 49.8 KDa.

The commercially available mouse anti-FLAG monoclonal primary antibody (Sigma F3165) used to probe FLAG-tagged monarch proteins expressed in DpN1 cells has been validated for western-blot against the FLAG immunogen sequence DYKDDDDK, as documented on the manufacturer's website.

Eukaryotic cell lines

Policy information about cell lines

Cell line source(s)	Monarch butterfly DpN1 cells (Palomares, L. A., Joosten, C. E., Hughes, P. R., Granados, R. R. & Shuler, M. L. (2003). Novel insect cell line capable of complex N-glycosylation and sialylation of recombinant proteins. Biotechnology progress 19, 185-192).
Authentication	Monarch butterfly DpN1 cells were obtained from the Reppert laboratory, who authenticated them via western blotting of several core clock proteins using monarch specific antibodies (Zhu H, Sauman I, Yuan Q, Casselman A, Emery-Le M, Emery P, Reppert SM. (2008). Cryptochromes define a novel circadian clock mechanism in monarch butterflies that may underlie sun compass navigation. PLoS Biol 6, e4). Sequencing of the period gene in the Merlin laboratory was used to confirm that the cells used were monarch cells.
Mycoplasma contamination	The cell line was not tested for mycoplasma contamination.
Commonly misidentified lines (See ICLAC register)	None.

Animals and other organisms

Policy information about studies involving animals; ARRIVE guidelines recommended for reporting animal research

Laboratory animals	Monarch butterflies (Danaus plexippus)/wild-type, Cry1-/-, Cry2-/- strains, male and female adults, two to six weeks old.
Wild animals	Wild fall migrants (males and females of unknown age) were captured in Dallas and College Station and brought back to the laboratory in glassine envelopes. They were checked for the presence of the protozoan parasite Ophryocystis elektroscirrha, and all monarch tested positive were immediately frozen at -80C. The remaining monarchs were fed a 25% honey solution before being housed in glassine envelopes in a 11-hours light:13-hours dark cycle set to the prevailing lighting conditions.
Field-collected samples	The study did not involved samples collected from the field.
Ethics oversight	No ethical approval or guidance was required as the monarch butterfly is an invertebrate.

Note that full information on the approval of the study protocol must also be provided in the manuscript.
