# Supplementary material for: Cryptochrome 1 mediates light-dependent inclination magnetosensing in monarch butterflies
Source: Nat Commun. 2021 Feb 3;12:771. doi: 10.1038/s41467-021-21002-z (PMC7859408; doi:10.1038/s41467-021-21002-z)
Supplement: Supplementary file 4 — Description of Additional Supplementary Files [file 41467_2021_21002_MOESM4_ESM.pdf]

Description of Additional Supplementary Files

Supplementary Movie 1. Movie of a monarch showing a positive light-dependent magnetic response to a reversal of the ambient magnetic inclination as seen in *dpCry1*$^{+/+}$, *dpCry2*$^{+/+}$, *dpCry2*$^{-/-}$ and laboratory-raised wild-types with clear painted antennae or eyes.

Supplementary Movie 2. Movie of a monarch showing no magnetic response to a reversal of the ambient magnetic inclination under different lighting conditions as seen in *dpCry1*$^{-/-}$ and laboratory-raised wild-types with black painted antennae or eyes.
