# Supplementary material for: Cryptochrome 1 mediates light-dependent inclination magnetosensing in monarch butterflies
Source: Nat Commun. 2021 Feb 3;12:771. doi: 10.1038/s41467-021-21002-z (PMC7859408; doi:10.1038/s41467-021-21002-z)
Supplement: Supplementary file 2 — Peer Review File [file 41467_2021_21002_MOESM2_ESM.pdf]

Reviewer #1 (Remarks to the Author):

In this manuscript Wan and colleagues explore the genetic basis of the magnetic sense in Monarch Butterflies. Specifically, they test the radical pair based theory of magnetoreception and the role of the cryptochrome proteins. They begin with the development of a new behavioural assay which assesses the activity of tethered Monarch's in response to changes in the magnetic field inclination. This assay appears to be astonishingly robust which is in stark contrast to all other behavioural assays that interrogate the magnetic sense. Employing this assay they report that magnetically induced hyperactive behaviour in both lab raised and migrant butterflies. They further report that magnetically induced hyperactive behaviour requires the presence of Cry1, but not Cry2 as well as broad spectrum or UV light. Their Cry2 results are consistent with data that shows that Cry2 does not bind FAD, and is therefore is an unlikely candidate as a primary sensor. This result is highly important for the field.

All in all, this paper has the potential to be a classic but falls short because of inadequate controls. First, an absolutely pre-requisite for any study in the field of magnetoreception is the use of double-wrap coils. These control for heat or vibration which can result from generating magnetic fields with Helmholtz coils. Similarly, sound can be generated when coils are switched on – which is controlled for by double wrapped coils. Second, there is mounting data that magnetic behaviour (in both vertebrates and invertebrates) is disrupted by low intensity (nT) broad band radio frequencies. Such fields are abundant in the laboratory environment. There is no evidence that the behavioural assay reported here was shielded in any way shape or form with a Faraday cage. Third, it is essential that all experiments are done in a totally blinded fashion both to genotype and magnetic condition. Again this does not appear to have been done. As the aforementioned issues have not been addressed, persuading the community that their astonishingly robust behavioural results are real will be an uphill battle. Should this be rectified I would recommend immediate publication of the paper.

I would add the following further comments:

1. The authors have generated several new Cry transgenics in Monarch's which will be extremely valuable tools, however, they should be validated further by: (a) sequencing the mRNA; (b) quantitating the mRNA; and (c) quantitating the protein levels. The latter should be feasible given they have an antibody (so long as it binds to the N-terminus).
2. Was the camera used to record the activity of butterflies shielded? You would expect it to emit RF.
3. Is there an impariment in general activity levels in general in Cry1 mutants? This is important to quantitate because it could account for the phenotype they observe.

Reviewer #2 (Remarks to the Author):

First let me say that although I am not a behavioural biologist (or indeed any kind of biologist), I have read and reviewed a lot of papers on magnetic sensing, cryptochromes, radical pair mechanism and so on. I have reasonable confidence that I can judge this work reliably. In terms of the care with which the experiments appear to have been conducted, the quality of the data, the clarity with which the results are described, and the caution with which conclusions are drawn, this is easily amongst the top 10% of articles in this field. I have only minor comments, suggestions and questions.

Unlike many others in this field, Wan et al. are careful not to over-interpret their findings. Conclusions such as "It also provides the first evidence that the CRY1 protein is involved in the detection of vector direction (i.e. magnetic inclination), supporting its role in a geomagnetic compass" (lines 176-179), which do not claim that CRY1 is the compass sensor or even that it plays a photoreceptor role in magnetic sensing, are spot on. However, a couple of other statements are less cautious, especially when referring to the work of other authors. For example (lines 185-186), "Although monarch and human CRY2s have been shown to be light- and magneto-sensitive in the Drosophila cellular

environment (refs 5,9,10)" and (line 61), "The discovery that type 2 CRYs were magnetosensitive … (refs 5,9)". Despite the claims of the authors of these papers, their results do not show that CRY1s or CRY2s are light-sensitive or magneto-sensitive in Drosophila. If the results in these papers are reliable (and I have serious doubts about that), all they show is that CRYs are involved somehow in magnetic sensing - perhaps only as signal transducers downstream of a different magnetic sensor which might not even be a radical pair, a possibility that Wan et al. acknowledge in lines 205-206.

One point on which the authors might like to comment is the wavelength-dependence of the magnetic hyperactivity (MH) behaviour. The absorption spectrum of cryptochromes that contain FAD in the fully oxidised state that is essential for radical pair formation, have a strong absorption band in the blue centred at 450 nm which extends out to about 500 nm. The light from the green diodes (480-580 nm) should therefore result in some degree of radical pair formation in CRY1. Can the absence of MH under green light be interpreted as evidence for a photoreceptor with an absorption spectrum that extends less far into the green than does CRY? Or is this observation consistent with CRY1 as the photoreceptor and simply a result of the low level of photo-activation by the 480-500 nm component of the output from the green diodes?

Studies of the biological effects of weak magnetic fields are rarely convincing unless they meet two criteria. They should be fully blinded to avoid any possibility of unconscious bias on the part of those conducting the experiments and they require the use of double-wound coils to avoid artefacts from sources such as heating, vibrations, etc. A good description of this can be found in Kirschvink et al., J. R. Soc. Interface 7 (2010) S179-S191. The experiments described used neither of these precautions. However, in this case, I don't think this is a problem. As the authors say in the Reporting Summary: "Investigators were not blinded to group allocation during data collection and analysis. Blinding was not applied because the magnetic response in this work are quantitative and recorded using an infrared LED beam." The authors also seem to have checked carefully for temperature rises. If there is some other artefact responsible for the MH, then I can't see why it should be present under some experimental conditions and not others.

Minor points

Pedantically, the phrase "radical pair(s) of electrons" (lines 37-38, 49, 196) is a little unusual. While there is indeed an unpaired electron in each of the radicals in a radical pair, and the spin dynamics of these unpaired electrons is crucial for the mechanism, I don't think the "of electrons" adds much to the label "radical pair". The phrase might even be interpreted as a pair of electrons with fundamental or essential (= "radical") properties.

Line 39: singlet-triplet

Line 100: previously

Line 127: light-activatable

The black and purple colours in the figures are difficult to distinguish. I recommend using a lighter shade of purple.

Reviewer #3 (Remarks to the Author):

This is a very interesting and thorough study dissecting the involvement of Cry1 and Cry2 in monarch butterfly magnetoreception. The newly developed behavioural assay offers a convincing way to test the response of the butterflies to an inversion of the vertical component of the magnetic field at ambient, Earth-strength magnetic field intensities. The study convincingly demonstrates that Cry1, but

not Cry2, is involved in light-dependent magnetoreception, and that the receptors are located in the antennas and retina of the butterflies.

Specific comment:
Lines 87-90: This sentence is difficult to understand unless the details in the methods section are also read. Please be a bit more specific and include information on the 30-min adaptation and under what conditions the animals were held during the 5-min break.
The Y-axes in the irradiance curves should be photons s-1 cm-2 nm-1 (spectral irradiance per nm), unlike the total irradiance which is correctly given as photons s-1 cm-2)

Rachel Muheim

REVIEWER COMMENTS

Reviewer #1 (Remarks to the Author):

In this manuscript Wan and colleagues explore the genetic basis of the magnetic sense in Monarch Butterflies. Specifically, they test the radical pair based theory of magnetoreception and the role of the cryptochrome proteins. They begin with the development of a new behavioural assay which assesses the activity of tethered Monarch's in response to changes in the magnetic field inclination. This assay appears to be astonishingly robust which is in stark contrast to all other behavioural assays that interrogate the magnetic sense. Employing this assay they report that magnetically induced hyperactive behaviour in both lab raised and migrant butterflies. They further report that magnetically induced hyperactive behaviour requires the presence of Cry1, but not Cry2 as well as broad spectrum or UV light. Their Cry2 results are consistent with data that shows that Cry2 does not bind FAD, and is therefore is an unlikely candidate as a primary sensor. This result is highly important for the field.

All in all, this paper has the potential to be a classic but falls short because of inadequate controls. First, an absolutely pre-requisite for any study in the field of magnetoreception is the use of double-wrap coils. These control for heat or vibration which can result from generating magnetic fields with Helmholtz coils. Similarly, sound can be generated when coils are switched on – which is controlled for by double wrapped coils. Second, there is mounting data that magnetic behaviour (in both vertebrates and invertebrates) is disrupted by low intensity (nT) broad band radio frequencies. Such fields are abundant in the laboratory environment. There is no evidence that the behavioural assay reported here was shielded in any way shape or form with a Faraday cage. Third, it is essential that all experiments are done in a totally blinded fashion both to genotype and magnetic condition. Again this does not appear to have been done. As the aforementioned issues have not been addressed, persuading the community that their astonishingly robust behavioural results are real will be an uphill battle. Should this be rectified I would recommend immediate publication of the paper.

We would like to thank this reviewer for acknowledging the potential importance of our work, and for providing constructive criticisms to improve its quality with respect to using established standards in the field.

To address the reviewer concerns, we built a new double-wrapped coil system (of the same dimensions as the single-wrapped coils used originally) and a large (183 x 183 x 245 cm) six-sided faraday cage surrounding the flight simulator/coil system (see pictures below). We also shielded the camera used to record the butterfly activity inside the flight simulator by wrapping it with a copper mesh to prevent emission of possible radio frequencies. The current between the two copper wires in the double-wrapped configuration of the coil system was injected either in the same direction (test) or in opposite directions (control) using a manual switch

located outside of the faraday cage, and the lights including UV and green LEDs located on an arm (see pictures below) were also manually switched. For technical reasons and constraints inherent to our system, we were unable to automatize magnetic and lighting conditions, but results were recorded using an automated counter system. This precludes unconscious bias from the experimenter. We have however repeated the magnetic hypersensitivity experiments blindly to genotypes (i.e. *Cry1* and *Cry2* homozygous mutants and wild-type siblings), as requested.

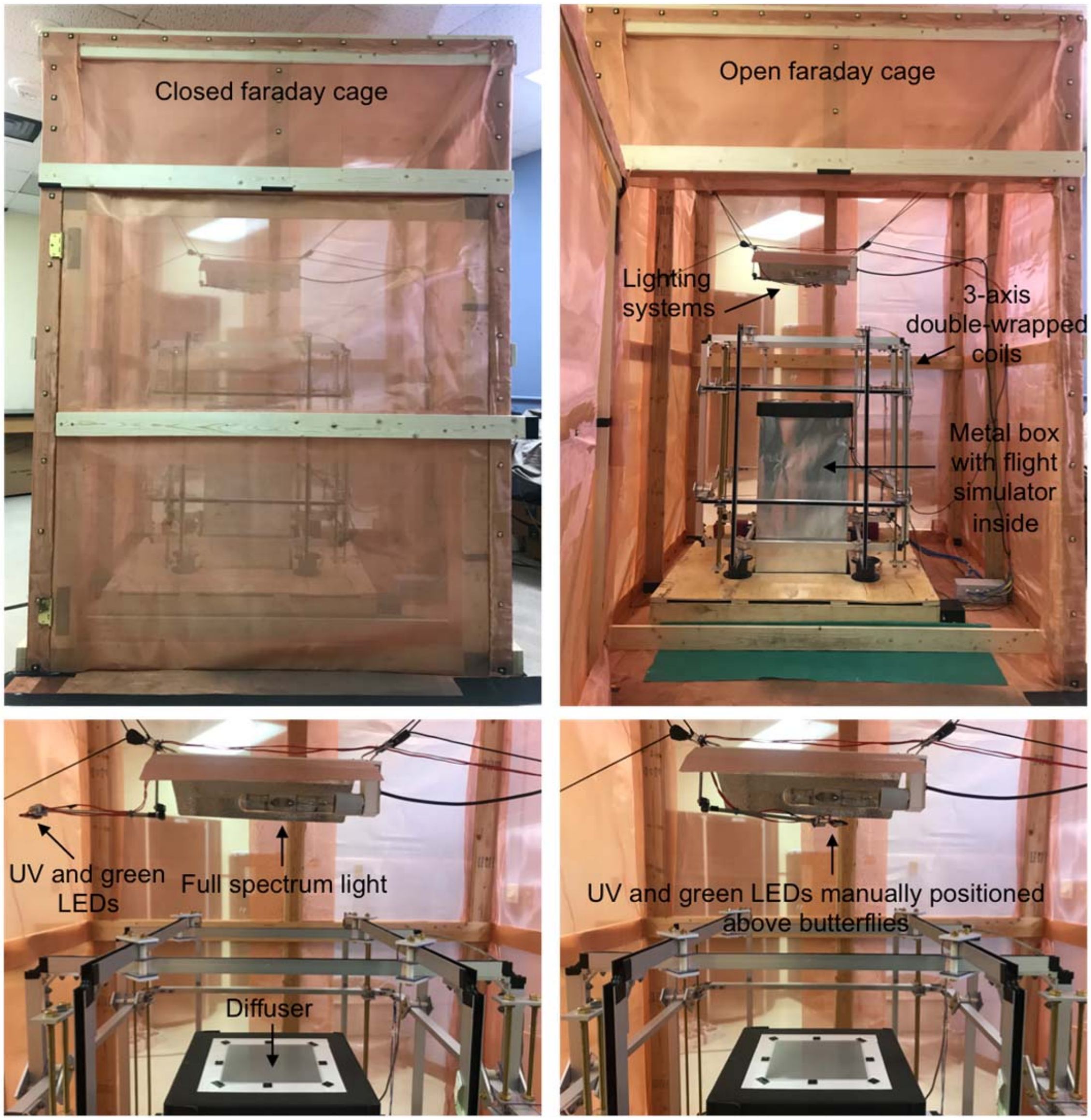

Cry1 and *Cry2* homozygous mutants and wild-type siblings were simultaneously raised and genotyped as larvae by members of the lab other than the experimenter involved in the magnetic behavioral responses. Adults of each genotype, which are phenotypically indistinguishable from one another, were randomly assigned a number by the head of the lab to ensure that both the experimenter and those

involved in genotyping would be blind to genotypes. Analysis was performed when all butterflies were behaviorally tested by sorting data according to genotypes. Importantly, the results we obtained using a double-wrapped coil surrounded by a large Faraday cage to blindly test monarchs (to genotypes) were in complete concordance with our original findings with non-blind tests. First, irrespective of the genotype and the lighting condition (full spectrum, darkness, UV-A/blue/Green), no response was found when the current was injected in opposite directions (control) in the copper wires in the double wrapped configuration. These data support the notion that the hyperactive behavior in response to a reversal of the magnetic field with our single-wrapped coil was not due to the heat or vibrations generated while switching on the coils, as these potential artifacts would also occur in the double-wrapped configuration when the current is injected in opposite directions (but no magnetic field is generated). Second, we found that 1) *Cry1*$^{+/+}$ responded to a reversal of the magnetic field only under full spectrum and UV-A/blue light while *Cry1*$^{-/-}$ did not (Extended Data Figure 3); and 2) *Cry2*$^{-/-}$ responded to a reversal of the magnetic field only under full spectrum and UV-A/blue light just like *Cry2*$^{+/+}$ (Extended Data Figure 7). The only notable difference between these data and the data reported with the single-wrapped coils (Figures 2 and 3) is the distribution of number of wingbeats exhibit by individual butterflies with fewer butterflies exhibiting greater than 30 to 40 wingbeats during reversal of the magnetic field and in the 10sec following return to ambient magnetic field. This difference might be the result of a different genetic background in these new batches of butterflies, as these lines underwent at least 5 generations of backcrossing to butterflies with a different background than those tested in Figures 2 and 3 (we bring wild-caught monarchs twice a year in our colony to prevent inbreeding).
We have described these new results in the main text lines 137-146 and lines 159-163, and added in Supplementary information the corresponding legends (lines 34-44 and lines 77-87), as well as a method section lines 138-152.

I would add the following further comments:

1. The authors have generated several new Cry transgenics in Monarch's which will be extremely valuable tools, however, they should be validated further by: (a) sequencing the mRNA; (b) quantitating the mRNA; and (c) quantitating the protein levels. The latter should be feasible given they have an antibody (so long as it binds to the N-terminus).

The reviewer is correct that we have generated a new monarch *Cry1* mutant, and we agree that reporting its characterization in as much depth as possible will be valuable. The second monarch mutant used in this study, *Cry2*, was previously generated (Merlin *et al*, *Genome Research*, 2013, 23:159-68), and part of its characterization has already been reported, but we nevertheless tried to complete it as requested.
For the *Cry1* mutant, we had identified that it consisted of a 2-bp deletion by sequencing the mutated allele, and now report the chromatograph in Extended Data

Figure 1a. As suggested, we also quantified the relative *Cry1* mRNA expression levels in brains of wild-type and homozygous mutant monarchs and reported the results in Extended Data Figure 1c. We found that *Cry1* was expressed in homozygous mutants at only about 10% of the wild-type levels, suggesting that the mutant RNA may undergo nonsense-mediated mRNA decay. If this is the case, one would expect no protein expression in the mutant. Because the only western blot-grade antibody available against monarch *Cry1* (dpGP37; Zhu *et al*, *PLoS Biol*, 2008, 6:e4) has been raised against the full length protein and not just the N-terminus, it would not allow us to ascertain whether it recognizes a truncated protein missing more than 50% at the C-terminal end. As an alternative, we FLAG-tagged wild-type CRY1 and CRY1Δ2 mutant, expressed each of them in monarch DpN1 cells along with FLAG-PER (used as a positive control for transfection), and performed a western blot using an anti-FLAG antibody. We found that while wild-type CRY1 signal was detectable at the expected size (~50KDa), no CRY1Δ2 was detectable (and this could not be due to problems with transfections as PER was detected in both conditions). These data are reported in Extended Data Figure 1d. Together with the quantitation of *Cry1* mRNA levels in *Cry1* mutants, these results suggest that the homozygous *Cry1* mutant monarchs do not express a functional CRY1. A description of the characterization of *Cry1* mutant has now been added in the main text lines 110-118, and the corresponding methods have been added in Supplementary information lines 171-215.
For the *Cry2* mutant bearing a 4-bp deletion, as mentioned above, some of the characterization, *i.e.* the sequencing of the mRNA and validation of protein expression in wild-type and mutant monarchs had already been published (Merlin *et al*, 2013). V5-tagged full length and truncated CRY2 were expressed in *Drosophila* Schneider 2 cells, and both forms were detectable with a V5 antibody, suggesting that the truncated protein, which lacks the C-terminal domain containing the Trp tetrad, is likely expressed in *Cry2* monarch mutants. The quantification of the *Cry2* mRNA in brains of wild-type and *Cry2* mutant monarchs that we performed to complete the characterization of this mutant revealed no significant difference in the levels of *Cry2* expression. A chromatograph of the mutated allele at the targeted region and the quantification of the *Cry2* mRNA levels have now been included in Extended Data Figure 5, and the methods for quantification have been added in Supplementary information lines 171-186. The results have also been included in main text lines 155-158.

2. Was the camera used to record the activity of butterflies shielded? You would expect it to emit RF.

The camera used to record the monarchs' activity was not shielded in our set of experiments using the single-wrapped coil system, but was shielded with a copper mesh in the experiments performed for the revisions of this manuscript with the double-wrapped coils, as mentioned above. Given that our results for both *Cry1* and *Cry2* homozygous mutants and wild-type siblings are consistent regardless of the coil system used and whether the camera was shielded or not, if the camera emitted

radio frequencies, it would appear that these did not significantly affect the magnetic hyperactivity behavior tested and the interpretation of the results.

3. Is there an impariment in general activity levels in general in Cry1 mutants? This is important to quantitate because it could account for the phenotype they observe.

Thank you for commenting on this. We agree that it is an important control to show, and we have now quantified general activity levels between *Cry1* homozygous mutants and wild-type siblings by measuring the distance flown by individuals of each genotype over the course of several days using a flight mill. We found that on each of the three days tested, both *Cry1*$^{+/+}$ and *Cry1*$^{-/-}$ were active only during the day (as anticipated for a diurnal species), and flew distances that were not significantly different from one another (Extended Data Figure 4). These results support the idea that the overall activity levels of *Cry1*$^{-/-}$ are not impaired, and thus not be the cause of the lack of magnetic responses we observed in *Cry1*$^{-/-}$. We added a sentence lines 146-148 in the main text, as follows: “Importantly, the lack of MH observed in *dpCry1*$^{-/-}$ monarchs was not due to impaired general activity levels, as these mutants flew as actively in a flight mill as their wild-type siblings over three days (Extended Data Fig. 4; $p \geq 0.06$, two-tailed Mann-Whitney *U*-test)”. The corresponding methods are described in the method section in supplementary information lines 154-169.

Reviewer #2 (Remarks to the Author):

First let me say that although I am not a behavioural biologist (or indeed any kind of biologist), I have read and reviewed a lot of papers on magnetic sensing, cryptochromes, radical pair mechanism and so on. I have reasonable confidence that I can judge this work reliably. In terms of the care with which the experiments appear to have been conducted, the quality of the data, the clarity with which the results are described, and the caution with which conclusions are drawn, this is easily amongst the top 10% of articles in this field. I have only minor comments, suggestions and questions.

We thank this reviewer for appreciating the impact of the work presented in this manuscript, and for providing very useful feedback to further improve its quality. All comments have been addressed point by point below.

Unlike many others in this field, Wan et al. are careful not to over-interpret their findings. Conclusions such as "It also provides the first evidence that the CRY1 protein is involved in the detection of vector direction (i.e. magnetic inclination), supporting its role in a geomagnetic compass" (lines 176-179), which do not claim that CRY1 is the compass sensor or even that it plays a photoreceptor role in magnetic sensing, are spot on. However, a couple of other statements are less cautious, especially when referring to the work of other authors. For example (lines 185-186), "Although monarch and human CRY2s have been shown to be light- and magneto-sensitive in the Drosophila cellular environment (refs 5,9,10)" and (line 61), "The discovery that type 2 CRYs were magnetosensitive … (refs 5,9)". Despite the claims of the authors of these papers, their results do not show that CRY1s or CRY2s are light-sensitive or magneto-sensitive in Drosophila. If the results in these papers are reliable (and I have serious doubts about that), all they show is that CRYs are involved somehow in magnetic sensing - perhaps only as signal transducers downstream of a different magnetic sensor which might not even be a radical pair, a possibility that Wan et al. acknowledge in lines 205-206.

We have rephrased several statements throughout the manuscript to more cautiously describe the findings pertaining to work of other authors and tone down one of our interpretation, as follows:

Lines 61-62: "The discovery that type 2 CRYs were magnetosensitive…" was replaced by "The discovery that type 2 CRYs mediated light-dependent magnetosensing…".

Lines 214-215: "Although monarch and human CRY2s have been shown to be light- and magneto-sensitive in the *Drosophila* cellular environment (refs 5,9,10)" was modified as follows, "Although monarch and human CRY2s have been shown to mediate light-dependent magnetosensitivity in the *Drosophila* cellular environment (refs 5,9,10)".

Lines 216-218: "Studies in the mammalian suprachiasmatic nucleus, the brain structure harboring the master circadian clock, found no evidence of light- or magneto-sensitivity for mammalian CRYs,…" was replaced by "Studies in the mammalian suprachiasmatic nucleus, the brain structure harboring the master circadian clock, found no evidence of mammalian CRYs' involvement in magnetosensing,…".

Lines 219-221: "The use of full-body loss-of-function mutants in our *in vivo* study unambiguously demonstrates that in the proper cellular environment monarch CRY2 is not magnetosensitive*…"* was replaced by *"* The use of full-body loss-of-function mutants in our *in vivo* study unambiguously demonstrates that in the proper cellular environment monarch CRY2 does not play a role in magnetosensing*…".*

One point on which the authors might like to comment is the wavelength-dependence of the magnetic hyperactivity (MH) behaviour. The absorption spectrum of cryptochromes that contain FAD in the fully oxidised state that is essential for radical pair formation, have a strong absorption band in the blue centred at 450 nm which extends out to about 500 nm. The light from the green diodes (480-580 nm) should therefore result in some degree of radical pair formation in CRY1. Can the absence of MH under green light be interpreted as evidence for a photoreceptor with an absorption spectrum that extends less far into the green than does CRY? Or is this observation consistent with CRY1 as the photoreceptor and simply a result of the low level of photo-activation by the 480-500 nm component of the output from the green diodes?

Thank you for bringing up this important point. Whether the lack of MH response to a reversal of the magnetic inclination in our experiments with the green diodes could be interpreted as evidence for a photoreceptor other than CRY1 (such as an opsin) or evidence that CRY1 is the sole photoreceptor involved would depend, as rightly pointed out, on whether the light emitted between 480-500 nm would be sufficient to generate radical pair formation of CRY1. Given that we do not have this information at this time, it is not possible to favor one hypothesis over the other. However, we feel that it is an important point to bring into the discussion, and we have now included a paragraph lines 233-245 to that effect. It reads as follows: "Despite the central role that light-sensitive CRYs play in light-dependent magnetoreception, the notion that they function as *bona fide* photo-magnetoreceptors is still under debate[33]. The absence of magnetic hypersensitivity to a reversal of the ambient magnetic field under green light emitting between 480 and 580 nm in our assay may provide some insights. Indeed, the absorption spectra of type 1 CRYs bound to fully oxidized FAD necessary for radical pair formation extends to ~ 500 nm[34]. The lack of magnetic response under green light could be due to insufficient photo-activation to give rise to radical pair formation in CRY1 between 480 and 500 nm. However, if there was sufficient photo-activation of CRY1, the lack of magnetic responses in our experiments could be interpreted as evidence for another photoreceptor in light-dependent magnetoreception with an absorption

spectrum that extends less far into the green than does CRY1s. A UV opsin would be an obvious candidate. The potential involvement of opsins in magnetoreception is not without precedent, as direct interaction between a member of the opsin family and the avian type 4 CRY has recently been reported[35], and could be genetically tested in the monarch".

Studies of the biological effects of weak magnetic fields are rarely convincing unless they meet two criteria. They should be fully blinded to avoid any possibility of unconscious bias on the part of those conducting the experiments and they require the use of double-wound coils to avoid artefacts from sources such as heating, vibrations, etc. A good description of this can be found in Kirschvink et al., J. R. Soc. Interface 7 (2010) S179-S191. The experiments described used neither of these precautions. However, in this case, I don't think this is a problem. As the authors say in the Reporting Summary: "Investigators were not blinded to group allocation during data collection and analysis. Blinding was not applied because the magnetic response in this work are quantitative and recorded using an infrared LED beam." The authors also seem to have checked carefully for temperature rises. If there is some other artefact responsible for the MH, then I can't see why it should be present under some experimental conditions and not others.

Thank you for these comments. To satisfy the requests by reviewer 1 and the editor, we have now repeated the magnetic hypersensitivity experiments with both *Cry1* and *Cry2* homozygous mutants and wild-type siblings in a blind fashion with respect to genotypes, using a newly built double-wrapped coil system (to control for possible artefacts while switching the coils) surrounded by a large six-sided faraday cage. Results from these experiments, which are presented in Extended Data Figure 3 and Extended Data Figure 7, match those obtained with the single-wrapped coil system. First, irrespective of their genotypes, none of the butterflies responded to the coils being switched on when the current was injected in opposite directions, eliminating the possibility that a behavioral response was caused by vibrations or heat. Second, we found that 1) *Cry1*$^{+/+}$ responded to a reversal of the magnetic field only under full spectrum and UV-A/blue light while *Cry1*$^{-/-}$ did not (Extended Data Figure 3); 2) *Cry2*$^{-/-}$ responded to a reversal of the magnetic field only under full spectrum and UV-A/blue light just like *Cry2*$^{+/+}$(Extended Data Figure 7). The only notable difference between these data and the data reported with the single-wrapped coils (Figures 2 and 3) is the distribution of number of wingbeats exhibit by individual butterflies with fewer butterflies exhibiting greater than 30 to 40 wingbeats during reversal of the magnetic field and in the 10sec following return to ambient magnetic field. This difference might be the result of a different genetic background in these new batches of butterflies, as these lines underwent at least 5 generations of backcrossing to butterflies with a different background than those tested in Figures 2 and 3 (we bring wild-caught monarchs twice a year in our colony to prevent inbreeding).
We have described these new results in the main text lines 137-146 and lines 159-163, and added in Supplementary information the corresponding legends (lines 34-44 and lines 77-87), as well as a method section lines 138-152.

Minor points

Pedantically, the phrase “radical pair(s) of electrons” (lines 37-38, 49, 196) is a little unusual. While there is indeed an unpaired electron in each of the radicals in a radical pair, and the spin dynamics of these unpaired electrons is crucial for the mechanism, I don’t think the “of electrons” adds much to the label “radical pair”. The phrase might even be interpreted as a pair of electrons with fundamental or essential (= “radical”) properties.

We have now removed “of electrons” lines 37, 49, and 225.

Line 39: singlet-triplet

The typo has been corrected.

Line 100: previously

This has been corrected line 103.

Line 127: light-activatable

The change has been made line 149.

The black and purple colours in the figures are difficult to distinguish. I recommend using a lighter shade of purple.

Thank you for pointing this out. We have now used a lighter shade of purple in all figures.

Reviewer #3 (Remarks to the Author):

This is a very interesting and thorough study dissecting the involvement of Cry1 and Cry2 in monarch butterfly magnetoreception. The newly developed behavioural assay offers a convincing way to test the response of the butterflies to an inversion of the vertical component of the magnetic field at ambient, Earth-strength magnetic field intensities. The study convincingly demonstrates that Cry1, but not Cry2, is involved in light-dependent magnetoreception, and that the receptors are located in the antennas and retina of the butterflies.

We thank this reviewer for assessing the work presented in this manuscript, and providing the specific comments below.

Specific comment:
Lines 87-90: This sentence is difficult to understand unless the details in the methods section are also read. Please be a bit more specific and include information on the 30-min adaptation and under what conditions the animals were held during the 5-min break.

We have now reworded this sentence (lines 89-93) to clarify this point, as follows (additions underlined):"Each individual was <u>acclimated in darkness for at least 30 min prior to the test before being</u> subjected to 2 min of constant local ambient magnetic inclination (AMI; control) under white light. <u>A</u>fter 5 min <u>of break</u> under the same lighting and magnetic conditions, <u>the same individual was subjected again</u> to 2 min of ambient magnetic field but during which the inclination was reversed for 10 sec starting 20 sec after AMI was initiated (RAMI; Fig. 1d)".

The Y-axes in the irradiance curves should be photons s-1 cm-2 nm-1 (spectral irradiance per nm), unlike the total irradiance which is correctly given as photons s-1 cm-2).

Thank you for catching this. We have now changed the y-axes of Figures 1c and 2a to photons s-1 cm-2 nm-1.

Rachel Muheim

Reviewer #1 (Remarks to the Author):

The authors have done an admirable job at addressing the points I raised. No doubt these experiments were challenging given the current working restrictions, which I further appreciate. The manuscript is much stronger than the initial submission, and in my view ready for publication.

I strongly encourage the authors to continue to use blinded protocols, double wrapped coils, and a Faraday cage in the future. With their powerful assay, I look forward to seeing their results in the coming years. They are in a position to do a bunch of real cool experiments.

Reviewer #2 (Remarks to the Author):

The authors have satisfactorily dealt with all the comments I had on the original version of their manuscript.

Reviewer #3 (Remarks to the Author):

I am happy with the revised version of the manuscript and have no further questions.

While in my opinion (and see also comment by reviewer #2) the original data left little, if any, room to be misinterpreted due to systematic artifacts in the experimental setup because the magnetic coils were not doubly wrapped and the setup was not shielded, it is nevertheless good to see that neither the introduction of shielding nor a doubly-wrapped coil changed the outcome of the study. Altogether, this makes the study even more convincing now!

REVIEWERS' COMMENTS

We are grateful to the three reviewers for taking the time to provide high quality and critical reviews that have helped us improve the quality of the manuscript.

Reviewer #1 (Remarks to the Author):

The authors have done an admirable job at addressing the points I raised. No doubt these experiments were challenging given the current working restrictions, which I further appreciate. The manuscript is much stronger than the initial submission, and in my view ready for publication.

I strongly encourage the authors to continue to use blinded protocols, double wrapped coils, and a Faraday cage in the future. With their powerful assay, I look forward to seeing their results in the coming years. They are in a position to do a bunch of real cool experiments.

Response: We agree that despite being challenging, the additional experiments we performed have strengthened the work in ways that not only benefit this manuscript but also how we will conduct future experiments (for which we will stick to blinded protocols, double wrapped coils, and a Faraday cage). Thank you for helping us tightening the work!

Reviewer #2 (Remarks to the Author):

The authors have satisfactorily dealt with all the comments I had on the original version of their manuscript.

Response: We thank this reviewer for making great suggestions that have helped us adjust some of the arguments and enrich the discussion.

Reviewer #3 (Remarks to the Author):

I am happy with the revised version of the manuscript and have no further questions.

While in my opinion (and see also comment by reviewer #2) the original data left little, if any, room to be misinterpreted due to systematic artifacts in the experimental setup because the magnetic coils were not doubly wrapped and the setup was not shielded, it is nevertheless good to see that neither the introduction of shielding nor a doubly-wrapped coil changed the outcome of the study. Altogether, this makes the study even more convincing now!

Response: Thank you for the kind words and help with the manuscript.
